# Health and wellbeing experiences of women informal workers during the COVID-19 pandemic: a qualitative systematic review

**DOI:** 10.1186/s12889-025-25728-z

**Published:** 2025-11-28

**Authors:** Siti Aishah Ibrahim, Iman Ghosh, Suat Yan Lai, Bronwyn Harris, Abimbola Ayorinde

**Affiliations:** 1 Institute for Global Pandemic Planning, University of Warwick, Coventry, CV4 7AL UK; 2 Warwick Medical School, University of Warwick, Coventry, UK; 3 Centre for Evidence and Implementation Science, University of Birmingham, Birmingham, UK; 4 Gender Studies Programme, Faculty of Arts and Social Sciences, Universiti Malaya, Kuala Lumpur, Malaysia; 5 Centre for Global Health, University of Warwick, Coventry, UK

**Keywords:** Informal employment, Women’s health, COVID-19, Social determinants of health, Intersectionality

## Abstract

**Background:**

Lacking social safety nets, women informal workers were adversely affected during the COVID-19 pandemic, particularly with their over-representation in hard-hit sectors and poor prospect of alternative employment. Differential needs of women informal workers during the pandemic must be explored to address health inequalities and inform pandemic preparedness. This systematic review aimed to synthesise qualitative evidence on the health and wellbeing experiences of women informal workers during the recent pandemic.

**Methods:**

MEDLINE, Web of Science, Scopus and PsycINFO were systematically searched in November 2024, along with supplementary searches in Google Scholar and WHO COVID-19 research database. Citation tracking of included studies and grey literature search were also performed. Eligible studies explored first-person accounts of women informal workers about their health and wellbeing during the pandemic, and collected data using interviews, focus groups, or observations. Quality assessment of included studies was completed using Mixed Methods Appraisal Tool. Drawing from the concept of determinants of health, thematic approach was used to synthesise findings, taking a holistic perspective of health and wellbeing experiences during the pandemic.

**Results:**

Fifty-three studies were included, majority conducted in low-and middle-income countries. Studies explored the experiences of women in various forms of informal work including sex workers, domestic workers, traders/vendors and home-based workers. Four themes were generated: (1) exacerbation of existing vulnerabilities; (2) negotiation of risks and resilience; (3) interconnectedness of health and wellbeing stressors; and (4) variable experiences across social locations. Financial strain from livelihood loss was the primary stressor during the pandemic, and through cascading effect impacted other health and wellbeing domains. Stressors experienced were deeply interrelated and shaped by structural determinants – socioeconomic, political and cultural – as well as pandemic response policies. The experiences of women informal workers were mediated by intersecting social identities such as occupation type, caste/class and migration status, which influenced both their vulnerabilities and capacities to respond, resulting in diverse and unequal outcomes.

**Conclusions:**

The COVID-19 pandemic has taken a toll on the health and wellbeing of woman informal workers. In the groundwork for future development and pandemic planning, equity-centred approach informed by intersectionality must be applied.

**Supplementary Information:**

The online version contains supplementary material available at 10.1186/s12889-025-25728-z.

## Background

Informal employment involves “all remunerative work (i.e. both self-employment and wage employment) that is not registered, regulated, or protected by existing legal or regulatory frameworks, as well as non-remunerative work undertaken in an income-producing enterprise” [1]. Extremely heterogenous, informal work encompasses a broad range of production and employment forms. Informal workers however share one thing in common: they are all working with minimal social protection [1]. This therefore leaves workers without any guarantee of income security, particularly in cases of unemployment, sickness, invalidity, or a critical period like the pandemic.

Women make up a large share of informal workers in the global south, with over 90% of women workers informally employed in Southern Asia and sub-Saharan Africa [2]. Given the lack of access to formal job opportunities in these developing regions, informal work serves as the dominant mode of employment as well as a safety net providing a source of income for women [2]. Within informal employment, a hierarchy of earning levels and gender segmentation exists – with women disproportionately concentrated in the bottom tier where productivity and income are low [3]. Even prior to the global outbreak of coronavirus disease 2019 (COVID-19), women informal workers were already earning less than their men counterparts with a wider gender wage gap than observed in formal employment [4]. The recent pandemic heightened these disparities, as women were overrepresented in hard-hit sectors such as manufacturing and retail [5, 6], and faced greater struggle securing alternative livelihoods [7]. Additionally, women informal workers faced heightened exposure to gender-based violence during the pandemic. Reports of police harassment including extortion, confiscation of goods, and physical violence were common, often justified under the guise of enforcing COVID-19 protocol [8, 9]. Within domestic spaces, lockdowns intensified contact with perpetrators, contributing to a surge of domestic violence cases [10]. The amplified burden of unpaid care work amidst the pandemic further compounded their stress and vulnerability [5].

COVID-19 pandemic has significantly compromised the health and wellbeing of 740 million women whose livelihoods depend on the informal economy [2]. Understanding the factors that shaped their experiences during this crisis is essential to safeguard against widening inequalities. Whilst a growing body of literature has explored the experiences of women working informally, much of it focused on single-country contexts [11–13] or specific occupational groups [14, 15]. Our systematic review addresses this gap by drawing together qualitative evidence on the health and wellbeing experiences of women informal workers across diverse settings during the COVID-19 pandemic. By exploring the depth and nuance embedded within qualitative data, the review elucidates factors relevant to their experiences that may be obscured by quantitative methodologies [16]. In doing so, we offer contextually rich insights into the needs of this marginalised population and inform more equitable resource allocation in future pandemic responses. Our review resonates with the multilevel systems framework depicting the interactions between pandemic and diverse factors affecting worker health and wellbeing [17].

## Methods

A priori review protocol was registered on PROSPERO (CRD42023478526). The Preferred Reporting Items for Systematic Reviews and Meta-Analyses (PRISMA) 2020 statement [18] and the Enhancing Transparency in Reporting the Synthesis of Qualitative Research (ENTREQ) checklist [19] were followed to ensure transparency in reporting (Supplementary Material pp. 3–5).

### Search strategy

Primary literature source was a systematic search of four electronic databases chosen for optimal coverage: MEDLINE, Web of Science, Scopus, and PsycINFO. Databases were searched in November 2024 following consultation with an expert librarian. Search terms combined clusters of key words related to the research aim [20]. Searches were delimited to records published from 2020 onwards as COVID-19 was only declared a health emergency then [21]. We restricted results to English language because of limited access to translation resources and the potential for misinterpretation of nuanced content. We utilised features of Systematic Review Accelerator (SRA) software [22] to adapt the search syntax to different databases. Details of the search terms used are provided (Supplementary pp. 6–8).

Additionally, we perused the WHO COVID-19 research database and Google Scholar, limiting to the first 100 results from each supplementary source to ensure feasibility and focus on the most relevant studies [23]. We also conducted citation tracking of included studies facilitated by SRA [22] to identify additional literature. Grey literature was located through handsearching of theses repositories, as well as websites of relevant organisations.

### Eligibility criteria

Inclusion and exclusion criteria were specified using SPIDER tool [24] as guidance (Table 1). Eligible studies included accounts of health and wellbeing experiences during the COVID-19 pandemic from women informal workers, and used qualitative data collection methods of interviews, focus groups, or observations. We included mixed methods studies where qualitative component was reported separately, and studies involving other stakeholders if accounts of women informal workers (hereafter referred to as women) were distinguishable.

**Table 1:** Inclusion and exclusion criteria

	Definition of concept	Inclusion	Exclusion	Justification
Sample	For the purpose of this review, we have used the term woman to refer to cisgender woman aged at least 15 years old. The term informal work in the review referred to remunerative activities that are by law or in practice not regulated or protected by the government [1].	Studies where women informal workers as defined by the review are involved as research participants. Studies where research participants consist of women working informally as defined by the review for their source of livelihood. We explicitly recognised sex work as a form of employment and included studies with women involved in sex or sex-related services as their source of income. Studies with women in gig economy were also eligible.	Studies wherein women worked informally to supplement income from a formal employment. Studies wherein women only entered the informal economy during the pandemic. Studies with mixed participant populations that do not report data from women informal workers separately or studies where the occupational status of women was unclear.	We focused on women who have been sustaining their livelihoods in the informal economy as their circumstances may possibly be different from women who mainly earned their income in the formal economy or women who became informal workers as a result of the pandemic. This review foregrounds first-person voices and expressions of women informal workers, and hence excluded studies that only report surrogate perspectives. Although engagement in the gig economy may be covered by work agreement or platform terms, much of gig work tend to fall within the context of informality where workers face low social protection coverage.
Phenomenon of interest	COVID-19 pandemic is defined as the period in which COVID-19 was a global health emergency, circa 2020–2022.	Studies in the context of COVID-19 pandemic from any country and setting.	Studies in the context of other disease outbreaks or circumstances.	Due to the differing contexts in which the COVID-19 pandemic developed and the variable nature of pandemic responses adopted.
Study design		Qualitative studies and mixed methods studies where the qualitative findings are reported separately. Studies must collect data using interviews, focus groups, or observations.	Quantitative studies.	Aligned with the review aim, qualitative design avoids a reductive or mechanistic approach to ensure a focus on complex participants’ experiences.
Evaluation	Particular outcomes of interest include accounts of women regarding factors such as economic (including impact on job, income, and resources), social (including relationship with family and community), environmental (including housing, food security, and digital access), access to health and social care, health behaviours, and coping strategies.	Studies which make reference to health and wellbeing experiences as defined by the review.	Studies which explored other experiences not related to health and wellbeing as defined by the review during the COVID-19 pandemic. For example, studies exploring the experience of applying for a business capital or loan were excluded if health and wellbeing experiences of women informal workers were not reported.	This review focuses on the health and wellbeing experiences of women informal workers and the associated factors which may influence the experience.
Research type		Empirical research.	Non-empirical research such as reviews, editorials, discussion papers, opinion pieces, commentaries and theoretical work.	This review focuses on synthesising a body of knowledge on what has been documented empirically rather than conceptually or theoretically.
		Studies which full-text report are available in English language.	Studies reported in languages other than English.	Translation of non-English studies using interlingual tool may complicate interpretation of text and result in the loss of meaning.
		Studies published from 2020 onwards.	Studies published in 2019 or earlier.	COVID-19 was only declared a health emergency in 2020 [21].

Health and wellbeing experiences in this review were broadly conceptualised to encompass not only physical and mental health, but also the conditions that influence health outcomes. We drew from the holistic view of Dahlgren-Whitehead model of health determinants [25] and reflected on the interactions of factors to better understand the experiences of women informal workers in the context of COVID-19.

### Study screening

Search records were exported into EndNote reference management software and de-duplicated. Through a two-stage screening process, remaining records were independently screened by two reviewers (SAI and IG) using Rayyan web-based software [26]. Titles and abstracts were first screened followed by full-text articles to establish selection decisions. Consensus on study inclusion was reached through discussion among the two reviewers, but in cases of persistent disagreement, a senior author (AA) was consulted to make the final decision. If a study had resulted in multiple publications, only one publication which was the most relevant to our review aim was selected to avoid duplication or over-representation of data. Reasons for excluding studies after full-text screening were documented (Supplementary pp. 9–12).

### Data extraction and assessment of study quality

Descriptive data of included studies were extracted by SAI onto a piloted Microsoft Excel spreadsheet and checked by IG. Reporting quality was assessed by both SAI and IG using the Mixed Methods Appraisal Tool (MMAT) [27]. MMAT was chosen as it can concomitantly assess the quality of diverse types of empirical studies. We have not excluded or weighted any study based on quality criteria, but the assessment enhanced the overall rigor of the synthesis.

### Data synthesis

Text, tables and figures under the headings ‘Results’ or ‘Findings’ (or equivalent) that resulted from specified methods were used as data. Thematic synthesis was used, as it is an accessible method allowing synthesis across multiple studies whilst preserving a transparent trail. Facilitated by NVivo software, we followed three stages of thematic synthesis as described by Thomas and Harden [28] (Table 2).

**Table 2:** Thematic synthesis process

Stage		Description
1	Coding of data	The qualitative data of each included study were inductively coded line-by-line by SAI to capture their meaning and content. At least one code was assigned to all relevant segment, and multiple codes were applied where appropriate. Judgements were made during coding about whether data fitted into existing codes or required a new code to be generated. Working descriptions of codes were logged to clarify the thinking behind each. The final set comprised of 284 codes related to the health and wellbeing experiences of women informal workers during the COVID-19 pandemic. Example codes included ‘increasing debt’ and ‘labelled carriers of virus.
2	Developing descriptive themes	Similarities and differences between the codes were considered and related codes were identified. This process sometimes involved rearrangement and amalgamation of codes. The codes were then organised into a hierarchy of groups of codes, creating tree-like structures of broad descriptive themes which remain close to the findings of the primary studies. SAI and IG discussed the descriptive themes across this stage. There was iterative reference back to the primary studies from which the codes were derived to ensure coherence. The 18 descriptive themes related to women’s experiences of health and wellbeing during the recent pandemic, and some were informed by authors’ knowledge of social determinants of health.
3	Generating analytical themes	Links between descriptive themes were mapped and interpreted by SAI to generate four higher-order analytical themes that together address the review aim. Themes were discussed and refined amongst the wider research team (SAI, IG, SYL, BH, AA).

## Results

**Fig. 1:**
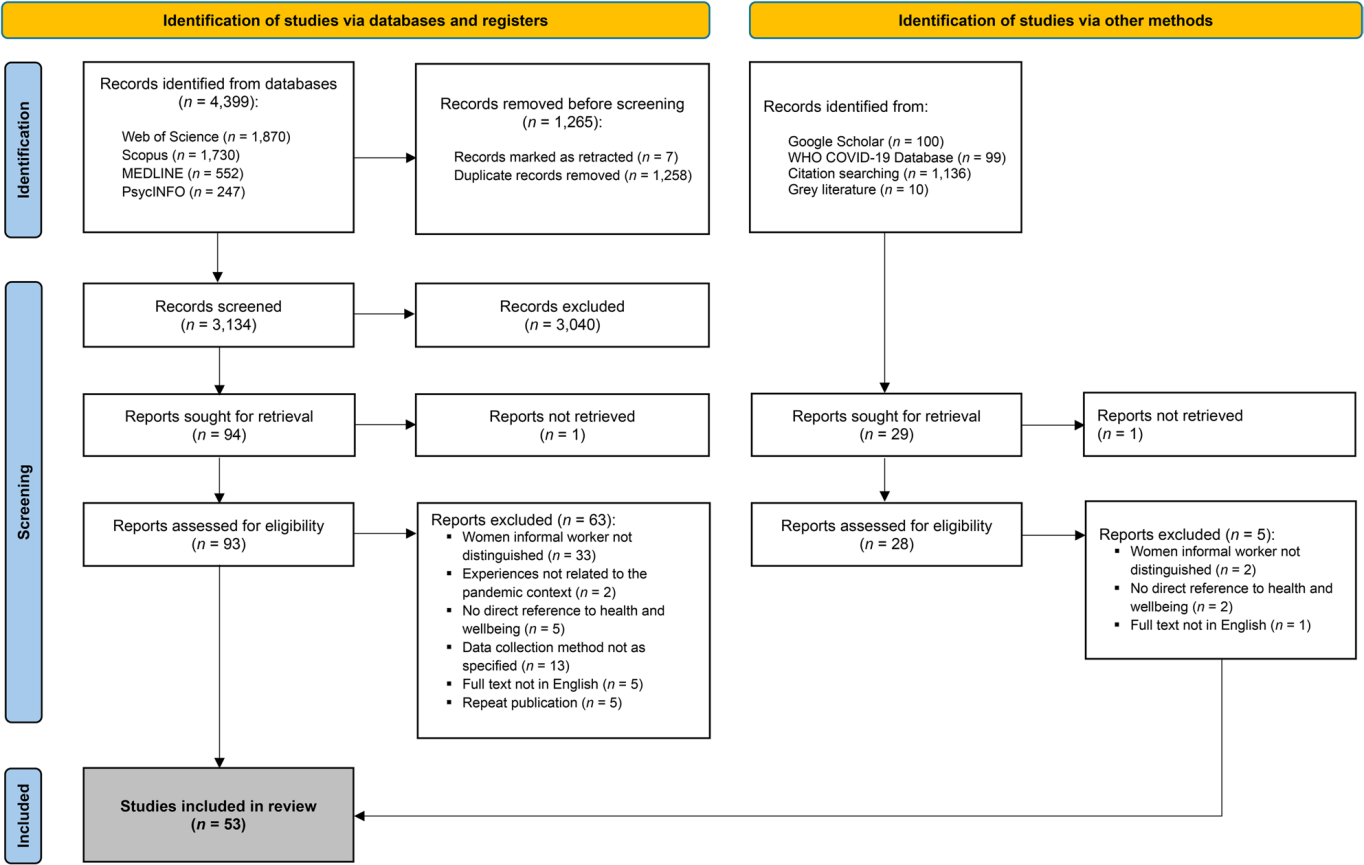
PRISMA flow diagram of study selection procedure

We screened 3,134 abstracts from primary database searches. Of these, 30 studies were included based on eligibility criteria. A further 23 studies were identified via other methods. This review is therefore based on 53 studies. PRISMA flow diagram outlining the selection procedure is presented in Fig. 1.

### Study characteristics

The included studies collectively represent the experiences of at least 1,465 women during the COVID-19 pandemic. Table 3 provides an overview of study characteristics. Studies were typically undertaken in low- and middle-income countries: India (*n* = 10) [34, 36, 39, 40, 42, 70, 71, 73, 75, 80], South Africa (*n* = 7) [56, 58, 60, 61, 64, 68, 72], Pakistan (*n* = 5) [31, 53, 62, 63, 81], Zimbabwe (*n* = 5) [51, 59, 65, 66, 74], Kenya (*n* = 3) [37, 55, 76], Ghana (*n* = 2) [30, 79], Uganda (*n* = 2) [57, 77], as well as 13 other countries with one study each [29, 32, 33, 35, 38, 41, 43, 45, 50, 52, 54, 69, 78]. Four studies were set in high-income countries namely Spain [46], Italy [47], United States of America (USA) [49], and Barbados [48], while two studies were conducted in multiple countries [44, 67]. The most common study design was qualitative (*n* = 41) [29, 31, 33–37, 39–41, 43–48, 50–66, 68, 69, 71, 72, 74, 78, 80, 81] and the remaining (*n* = 12) were mixed methods research with qualitative and quantitative descriptive components [30, 32, 38, 42, 49, 67, 70, 73, 75–77, 79].

**Table 3:** Characteristics of included studies. [29–81]

Author (Year, Country)	Methodology/Data collection method/Data analysis method	Study period	Study aim	Qualitative data sample of women informal workers	Key findings related to health and wellbeing during pandemic
Aantjes et al. (2022, Mozambique) [29]	Qualitative research/ Face-to-face and telephone interviews/ Thematic analysis	February – October 2021	To explore the economic, social and health impacts of COVID-19 responses on young people who sell sex, and the mitigation strategies taken by individuals, government and civil society organisations to help mitigate these impacts.	38 sex workers, 18–24 years old	• dwindling demand for sex work and loss of income; • food insecurity forced rationing on food and basic commodities; • heightened exposure to police brutality; • strategies for economic survival include relying on savings and better-off relatives; • use of telephone and social media to solicit clients.
Abrefa Busia et al. (2023, Ghana) [30]	Mixed methods research/ Face-to-face and telephone interviews, observation/ Thematic analysis	September 2020 – March 2021	To investigate the benefits that working mothers may experience from their work and family domains during the COVID-19 pandemic	14 women of mixed occupations, married, mothers, 27–64 years old	• women forced to get adequate rest and better sleep; • taking advantage of business opportunities to improve financial security; • positive mood due to more family time; • reduced house chores as family members helped out.
Agha et al. (2022, Pakistan) [31]	Qualitative research/ Face-to-face interviews/Thematic analysis	Not specified	To examine the impact of COVID-19 pandemic on women home-based workers women and how they survived the crisis	45 home-based workers (embroidery, tailoring and stitching), majority between 16–20 and 41–45 years old	• limited knowledge of COVID-19 and its damage at pandemic onset; • income loss and worsened struggle for survival; • coped by relying on family support, selling valuables and receiving government assistance.
Ajuwon & Ajuwon (2021, Nigeria) [32]	Mixed methods research/ Face-to-face interviews/ Thematic analysis	October 2020	To address the gap in knowledge about the knowledge, adherence to non-pharmaceutical interventions and effects of COVID-19 lockdown among traders in the informal sector	Market traders (number unspecified)	• knowledgeable about COVID-19 symptoms, means of transmission and prevention measures; • poor adherence to non- pharmaceutical interventions to prevent spread because of inconvenience and cost; • financial difficulties from reduction of income.
AleAhmad (2023, Iran) [33]	Qualitative research/Telephone interviews/Feminist intersectionality analysis	September 2021	To explore the embodied experiences of Kurdish female porters and how their lives and employment have been impacted by the COVID-19 crisis	1 porter, mother, sole breadwinner, 39 years old	• loss of only source of livelihood due to the pandemic resulting in deeper poverty; • financial security causing fear of death and anxiety about children’s health and future; • patriarchal culture perpetuates violence against women.
Arora & Majumder (2021, India) [34]	Qualitative research/ Face-to-face narrative interviews/ Thematic analysis	May – October 2020	To understand the lived experiences of migrant women workers during the pandemic and their narratives of gendered inequality	5 women of mixed occupations, migrants, 20–34 years old	• loss of livelihood translated into loss of decision making and mobility; • extra caregiving responsibilities; • increased domestic violence; • perilous journey of relocating to native villages; • mistreatment and humiliation during social distancing; • support from friendships with other migrant women.
Asriani et al. (2021, Indonesia) [35]	Qualitative research/ Online interviews, observation/ Gender analysis	May – June 2020	To understand the complexity of women’s experiences in the informal sector during the COVID-19 pandemic and its implications	28 women of mixed occupations	• belief of pandemic as a trial from God; • acceptance of pandemic conditions to maintain mental health; • lower exposure to COVID-19 than men as women stayed at home more often; • excessive and unreliable media information about pandemic causing anxiety; • increased burden of unpaid care; • less free time to rest and interact socially than men due to double burden.
Azeez E P et al. (2021, India) [36]	Qualitative research/ Online interviews/ Thematic analysis	May – July 2020	To explore the experience of women migrant labourers and their families during COVID-19 pandemic	19 domestic workers and cleaning staff, migrants, 29–56 years old	• loss of livelihood and resulting debt; • economic distress requiring adjustments of daily needs; • feelings of isolation and captivity; • disrupted access to services and facilities; • COVID-19 related anxiety and fear; • limited support from the government and other entities.
Babu et al. (2024, Kenya) [37]	Qualitative research/ Face-to-face and telephone interviews/ Thematic analysis	July – August 2020	To explore the ways in which female sex workers were affected by the COVID-19 control measures and the ways they coped with the hardships	47 sex workers, users of HIV prevention and treatment services, 18–45 years old	• police harassment and violence; • stigma, discrimination and social exclusion; • disrupted access and utilisation of health care services; • low negotiation power for safe sex; • poorer mental health; • intimate partner violence and strained relationship with children; • reduced number of clients and diminishing income.
Balampama et al. (2023, Tanzania) [38]	Mixed methods research/Face-to-face interviews/ Not specified	May 2018 – January 2022	To determine how the activities of female sex workers shifted over the course of the pandemic and to understand how they were affected economically	20 sex workers, mean age 28 years old	• concerns about exposure to COVID-19 but work is not conducive to taking precautions; • adapt to restrictions by using phones to contact clients; • low demands for sex affecting income, housing and food security; • income improvement seen in June-July 2021.
Banerjee & Wilks (2024, India) [39]	Qualitative research/ Face-to-face and telephone interviews/ Not specified	April – August 2020	To examine COVID-19 pandemic-related challenges both at work and at home for domestic workers	15 domestic workers, 20–55 years old	• loss of work and wages leading to difficulty paying for basic necessities; • feelings of uncertainty and powerlessness; • impact on affording healthcare; • heavier workloads and increased chores at home; • strained marital relationship; • fear of COVID-19 among employers heightening stigma and prejudice.
Barhoi & Dayal (2024, India) [40]	Qualitative research/ Face-to-face interviews, observation/ Thematic analysis	November – December 2021	To explore the plights of women temporary workers in tea gardens in Assam and to explore the impact of COVID-19 on their lives	26 tea plantation labourers, temporary workers, 20–61 years old	• lack of work during pandemic worsened livelihood as temporary workers; • unable to access relief money; • exploited to work longer hours for inadequate pay due to lack of other job opportunities; • increased domestic violence.
Belete (2020, Ethiopia) [41] [preprint]	Qualitative research/Telephone interviews/Thematic analysis	Not specified	To shed light on the untold effects of COVID-19 responses on the lives of commercial sex workers	12 sex workers	• job loss due to closure of bars and night clubs; • economic hardship affecting ability to afford food; • unable to access government support; • high risk of COVID-19 infection from nature of job; • unsafe sex practices; • increased societal stigma; • physical and verbal abuse by police officers.
Bhat et al. (2022, India) [42]	Mixed methods research/Telephone interviews/Thematic analysis	July 2020	To explore the impact of pandemic on the three aspects of work-life, household dynamics, and self-care/healthcare of domestic	40 domestic workers, survivors of domestic abuse, 18–62 years old	• fear, stress, acute anxiety and a sense of helplessness; • drastic reduction of income; • stigmatised as unhygienic; • lack of personal protective equipment and social distancing at workplace; • lack of rest from increased workload at home; • hunger and nutritional imbalance; • increased violence against women; • health misinformation; • difficulty accessing healthcare services.
Bishop & Laingoen (2024, Thailand) [43]	Qualitative research/Face-to-face interviews/Thematic analysis	March – April 2023	To explore the impact of COVID-19 lockdown on the lives of cis-gender female sex workers in Pattaya, and how they coped both practically and emotionally	10 sex workers, 26–35 years old	• closure of bars and entertainment venues led to loss of livelihood; • poor mental health; • coped by relocating to a bigger city or moving back with families; • shifted to street sex work or food vending which were largely non-viable; • relied financially on wider families, prior savings and remittances from foreign boyfriends; • none received financial aid from government but understood the limited funds of the government and proud of their self-resilience.
Boateng-Pobee et al. (2021, Multi-country) [44]	Qualitative research/Telephone interviews/ Not specified	March 2020 – June 2021	To shed light on the multiple and intersecting challenges faced by women informal traders in Accra, Ghana; Durban, South Africa and Nakuru, Kenya to both earn an income and care for their young children amidst an unprecedented global health, economic and care crisis	93 informal traders (street vendors, market traders and head porters), mothers and grandmothers caring for children 0–5 years old, 19–68 years old	• some informal traders were designated as essential workers and allowed to work but there was sustained loss of income; • head porters were more acutely affected with few customers in the market requiring their services; • traders in Durban faced harassment by police who were not aware of regulations allowing them to trade; • traders in Nakuru and Durban provided handwashing stations and sanitisers by government and non-governmental organisations respectively; • some brought their children to the markets despite fear of infection as homes were not safer options due to overcrowding and poor sanitation; • stressed about children’s nutritional needs and health.
Bossenbroek & Ftouhi (2021, Morocco) [45]	Qualitative research/Telephone interviews/ Not specified	March 2020	To explore how pandemic has affected female agricultural wageworkers and how they coped	25 agricultural labourers	• reduced work opportunities due to pandemic restrictions and low demand for labour; • financial problems resulting in cuts in basic provision and anxiety; • greater risk of COVID-19 infection at work due to lack of hygienic measures; • difficulty accessing governmental support programs; • relied on food donation from local municipal and non-governmental organisations; • coped by working secretly, diversifying income sources and borrowing money.
Burgos & del Pino (2021, Spain) [46]	Qualitative research/Telephone interviews/Thematic analysis	April 2020	To describe and understand the experience that women engaged in prostitution are undergoing in relation to the pandemic, confinement and their personal situations	11 sex workers. migrants, 23–63 years old	• women with COVID-19 symptoms were hidden from clients or moved to other places instead of given health check and treatment; • barrier in accessing health services due to illegal status; • working clandestinely after work venues were closed; • fear of COVID-19 infection but lack knowledge on protective behaviours; • greatest fear is not having money to pay rent and debt; • at high risk of COVID-19 infection due to work exposure and living conditions; • confinement increased social isolation and invisibility; • taken advantage by clients who demanded cheaper rate for service.
Cabras & Ingrascì (2022, Italy) [47]	Qualitative research/Ethnography/ Ethno-sociological approach	January 2020 – January 2021	To explore the challenges posed by the pandemic and the responses of female migrant sex workers	33 sex workers, migrants	• economic difficulties due to impossibility of working in the street; • mixed perception of COVID-19 risk; • well-informed about physical distancing regulations; • women respected curfew due to risk of being fined; • increased exploitation by cohabitant fiancés/pimps due to accumulated debt with them; • some coped by returning to home countries, some received support from non-governmental organisations or social services; • moved to online sex work for income but this could be riskier.
Cadogan-McClean & Cuffie (2023, Barbados) [48]	Qualitative research/ Online interviews and focus groups/ Thematic analysis	Not specified	To explore the experience of COVID-19 for women in the informal sector who provide wellness services for women and address the support they need	8 women of mixed occupations in wellness business	• loss of clients resulting in financial stress; • use of social media to conduct business online but faced technology anxiety; • physical health challenged by poor eating habits and weight gain during lockdown; • discomfort associated with wearing personal protective equipment; • stress, anxiety, and fear regarding the unknowns of the COVID-19 pandemic; • increased care responsibilities at home; • spiritual and self-care practices to manage stress; • received support from family, friends, and members of business community.
Callander et al. (2022, USA) [49]	Mixed methods research/Telephone and online interviews/ Thematic analysis	May – August 2020	To identify needs arising from the pandemic and opportunities for interventions to mitigate effects among sex work populations	8 sex workers, 23–54 years old	• discontinued work to reduce transmission risks and comply with public health orders; • loss of work opportunity resulted in financial loss; • food and housing insecurity; • lack of health insurance more pressing as access to health services was endangered; • forced to charge lower rates and engage in risky sexual practices; • negative effects on mental health and substance use; • resources activated to mitigate losses include social support, digital skills, and non-sex work employment; • sex workers of colour and those of lower socioeconomic status were more likely to experience negative effects.
Couto et al. (2022, Brazil) [50]	Qualitative research/ Face-to-face interviews/ Hermeneutic dialectic analysis	September – October 2020	To analyse coping strategies adopted by female sex workers in the face of stressors resulting from the COVID-19 pandemic	30 sex workers, majority 18–35 years old	• fear, anxiety and difficulties sleeping with the uncertainties of the future; • concern about personal and family support; • irritability and conflicts with co-workers and family members during confinement at home; • anxieties and insecurities generated by working conditions.
Crankshaw et al. (2023, Zimbabwe) [51]	Qualitative research/ Face-to-face interviews/Thematic analysis	March – May 2021	To explore the economic, social and health impacts of COVID-19 responses on gender and sexuality diverse young people who sell sex	22 sex workers, 16–24 years old	• reduced mobility and inaccessibility of previous workspaces influenced social and economic wellbeing; • some moved outside local area to seek clients and conceal work activities; • harassment and violence by law enforcement officials and clients; • high levels of food insecurity; • accepted reduced rates of payment due to economic impact; • those living in high density, impoverished areas were the most negatively impacted; • economic resilience by engaging in new enterprises.
de Silva (2022, Sri Lanka) [52]	Qualitative research/ Online interviews/ Thematic analysis	May – November 2021	To explore the challenges that women owners of micro- and small-scale businesses faced during the pandemic and how they leveraged digital technologies to overcome these challenges	20 micro- and small-scale entrepreneurs	• sharp decline in income resulting on financial difficulties; • Increased burden of care and household work; • exposure to COVID-19 due to proximity to customers and employees; • changes to business led to loneliness, frustration, hopelessness, and fears; • shift towards social media marketing and online transactions; • online communities as a safe space that provided emotional and social support.
Dogar et al. (2022, Pakistan) [53]	Qualitative research/ Face-to-face and telephone interviews/ Thematic matrices analysis	October 2020 – February 2021	To explore the impact of COVID-19 on women domestic workers and their families	54 domestic workers, majority between 21–40 years old	• difficulty accessing basic services during lockdown causing stress; • closure of cheaper private clinics limited access to health care services; • loss of livelihood and economic hardship; • borrowed money from family and friends for daily expoenses; • unable to get government cash assistance program due to selection process mechanism; • received help from charitable organisations and local community; • financial constraints causing psychological problems; • stress effect on marital relationship and subsequently domestic violence.
Fabbri (2022, Colombia) [54]	Qualitative research/ Face-to-face interviews/ Thematic analysis	June – July 2021	To explore the impact of COVID-19 on women involved in sex work	22 sex workers, Colombian natives and Venezuelan migrants, 18–59 years old	• mixed beliefs about COVID-19; • fear of contracting COVID-19 and spreading infection to older family members and children; • mixed views on COVID-19 vaccination; • not able to quarantine as need to make a living and pay off debt; • decrease in income with fewer clients; • inability to afford food and pay rent; • stigma and discrimination due to job and migrant status; • financial constraint and lack of legal documentation as barriers in healthcare service access; • adapt by changing work venues and doing alternative work outside sex industry; • social support from family members; • majority never received support or • assistance from any organization; • faith as a coping mechanism; • animosity between Colombian and Venezuelan women due to competition for clients.
Gichuna et al. (2020, Kenya) [55]	Qualitative research/ Telephone and video interviews/ Thematic analysis	April – May 2020	To highlight specific effects of COVID-19 and related restrictions on healthcare access for female sex workers living in informal settlements	117 sex workers, 16–46 years old, majority were breadwinners	• curfew and movement restrictions hindered access to health services; • fear and anxiety surrounding COVID-19 caused avoidance of health services for other health matters; • police brutality on those found breaking rules; • increased waiting time at healthcare facilities deterred access; • reproductive healthcare services neglected due to supply disruption and economic challenges facing women.
Hattar (2023, South Africa) [56]	Qualitative research/ Face-to-face interviews/ Thematic analysis	March 2023	To explore the experiences of female informal street traders during the COVID-19 pandemic	6 street traders, 32–54 years old	• pandemic-induced financial struggle; • finding alternative work to generate income; • weakened solidarity among women due to market competition and jealousy; • increased care burden; • police corruption; • insufficient government support; • relying on food parcels from church; • support from family, friends, and migrant community; • unique experiences and needs related to race/ethnicity, immigration status, and age.
King et al. (2023, Uganda) [57]	Qualitative research/ Telephone interviews/ Framework thematic analysis and interpretive description	May 2020 – July 2021	To understand the impact of COVID-19 pandemic and its associated restrictions on the daily lives of young sex workers and gauge their resilience	125 sex workers, mean age 21 years old	• layered dimensions of fear related to pandemic uncertainty and closure of social structures, but mainly due to financial and food security • feeling depressed and loneliness from social isolation, and stressed about children’s education; • heightened tensions at home, sometimes leading to violence; • social distancing enforced with arrests and violence by police • challenges accessing health services due to closure of facilities, transport issues, and lack of money; • mixed views on COVID-19 vaccine; • impact of pandemic changes over time as women learned from previous experiences.
Mapuranga et al. (2021, South Africa) [58]	Qualitative research/Face-to-face interviews/ Thematic analysis	Not specified	To examine the challenges faced by women survivalist entrepreneurs	35 survivalist entrepreneurs	• women fighting among themselves for strategic selling points; • decline in product demands; • confrontation with the police for not meeting COVID-19 regulatory rules.
Mavhandu-Mudzusi & Moyo (2022, Zimbabwe) [59]	Qualitative research/ Face-to-face interviews/Colaizzi’s method of data analysis	December 2020 – March 2021	To explore the effects of COVID-19 pandemic on female sex workers	10 sex workers, 31–45 years old	• emotional effect of forced disclosure of sexual practices to law enforcement agents; • police harassment; • significant loss in income; • anxiety and uncertainty related to income loss; • loneliness from home confinement; • inability to pay for food and rentals; • exposure to HIV and COVID-19 risky behaviours
Mbombo (2022, South Africa) [60]	Qualitative research/ Telephone and online interviews/ Not specified	September – October 2021	To explore the experiences of Black women as heads of families and how they were impacted by implemented governmental COVID-19 lockdown measures	5 women of mixed occupations, Black, head of families, 28–57 years old	• lack of access to sanitation and overcrowded household affecting COVID-19 protective behaviour; • loss of employment worsening poverty; • need to make a living so unable to comply with lockdown restrictions; • lack of formal banking affecting access to social relief grant; • inability to afford food and pay bills.
Mlambo & Masuku (2023 South Africa) [61]	Qualitative research/ Face-to-face interviews/ Not specified	August – November 2021	To shed new light on the sex industry by questioning the lack of government assistance for female sex workers during the COVID-19 pandemic and looking at their rights from a feminist perspective	11 sex workers	• evading law enforcement to continue working and generating income; • subjected to police violence and bribery; • insufficient government support; • civil society organisations as critical support structure; • changing methods of working including engaging in online or phone sex; • resorting to criminal activities to earn a living.
Mohmand et al. (2023, Pakistan) [62]	Qualitative research/ Face-to-face interviews/ Not specified	2021	To explore attitudes towards COVID-19 vaccination programmes among informal workers in the context of Lahore, Pakistan	Mixed occupations (number unspecified)	• most expressed beliefs that suggested mistrust of the COVID-19 vaccine; • popular belief that COVID-19 was a government plan to receive donation money; • some vaccinated due to concern for health but some felt forced to take it because of government directives; • views and willingness to get vaccinated affected by religious fatalism; • sectoral differences in pandemic perceptions and behaviour.
Mustafa et al. (2021, Pakistan) [63]	Qualitative research/ Telephone interviews and online focus groups/Thematic analysis	Not specified	To explore the impact of COVID-19 lockdown on micro-businesses owned by women borrowers of microfinance institutions and to provide policy suggestions to assist women entrepreneurs	7 entrepreneurs, borrowers of microfinance institutions, 30–55 years old	• decline in income with cessation of business activities; • difficulty affording basic needs and household expenses; • mental distress; • feeling of hopelessness; • lack of entrepreneurial skills enhanced by the pandemic.
Muswede & Sithole (2022, South Africa) [64]	Qualitative research/Telephone interviews/Thematic analysis	March – September 2020	To explore how migrant women personal functions of social media to deal with their situation at the height of the COVID-19 lockdown	17 mixed occupations, migrants, 23–47 years old	• loss of livelihood; • exclusion from COVID-19 related relief programmes and welfare benefits; • police corruption and collection of bribes to avoid arrest; • worry about being rendered illegal after unable to renew visas/permits due to lockdown; • adapted to home-based business following movement restriction • relied on social media for updates on lockdown and protocols; • social media messaging as platform for psychosocial support; • using social media to reach out to philanthropists and civic society for assistance.
Nhiwatiwa & Matanzima (2023, Zimbabwe) [65]	Qualitative research/ Face-to-face interviews, observation/ Thematic analysis	February – May 2021	To examine the impact of Covid-19-related lockdowns to the fisherwomen’s access to the Lake Kariba which impacted their fishing activity as a livelihood	50 fisherwomen, ≥40 years	• movement control resulted in precarity of fishing as a livelihood strategy; • continued fishing clandestinely to seek money and food; • engaged in alternative strategies to survive which were also precarious; food insecurity particularly for women with more dependents; • harassed, fined, and had fishing gear confiscated if caught breaking lockdown regulations; • lockdown in 2020 was more difficult than one in 2021.
Nyabeze et al. (2022, Zimbabwe) [66]	Qualitative research/ Face-to-face interviews, observation/ Not specified	Not specified	To assess the challenges faced by female sex workers during the COVID-19 lockdown and their resilience	10 sex workers	• loss of livelihood due to closure of bars and night clubs; • fear of online harassment; • fear of abuse from security officers; • breaching of curfew; • coped by changing times of operation, resorting to online sex trade, and starting small business; • increased alcohol and drug abuse; • exposure to HIV due to shortage of access to condoms; • exposure to environmental hazards.
Ogando et al. (2022, Multi-country) [67]	Mixed methods research/Telephone interviews/ Not specified	June – July 2020	To examine the contours of multidimensional crisis experienced by women in informal employment during the initial phase of the COVID-19 pandemic	Mixed occupations (number unspecified)	• increased care burden; • fear of contracting COVID-19 and spreading virus to other family members; • loss of work and earnings; • decreased food consumption particularly as women prioritised their children; • inability to afford food and pay bills; • dramatic debt increase from borrowing money at high interest rate; • uncertainty over the duration of lockdown measures caused anxiety and mental health issues; • insufficient and unevenly distributed cash transfer relief; • received food aid from worker organisations.
Oyebamiji (2023, South Africa) [68]	Qualitative research/ Face-to-face and telephone interviews/ Not specified	2021	To explore the experiences of Nigerian migrant women who operate in the informal economy in Durban	Mixed occupations (number unspecified), migrants from Nigeria, 20–50 years old	• loss of daily earnings for survival due to pandemic restrictions and lack of customers; • hunger and food insecurity; • feelings of stress and helplessness; • lack of relief and social support for migrants; • shifted to alternative business to secure livelihood; • struggling to recover financially from accumulated debt and non-operation.
Parlak et al. (2022, Turkey) [69]	Qualitative research/ Telephone interviews/Giorgi’s descriptive phenomenology	Not specified	To investigate the anxiety experiences of domestic workers who could not continue their working life during the COVID-19 pandemic	16 domestic workers, 30–42 years old	• anxiety with somatic symptoms related to contracting COVID-19; • some obsessions in taking precautions to avoid COVID-19; • increased feelings of isolation; • financial difficulty was one of the determinants of increased anxiety; • inability to pay rent, bills and loans; • relying on relatives to meet basic needs; • anxiety reduced among those who got support from the state; • effect on domestic relationship.
Samudyatha Kosambiya & Rathore (2024, India) [70]	Mixed methods research/Interviews and focus groups/Thematic analysis	April – May 2021	To document the impact of COVID-19 lockdown on women engaged in sex work and provision of sexually transmitted infection/reproductive tract infection/HIV preventive measures to them through targeted interventions and community-based organisations in Surat city	200 sex workers, majority between 30–39 years old	• continue to engage in sex work during lockdown due to financial reasons; • use of online sex work due to unavailability of places to work but there was apprehension about this method; • earning less pay per sex activity; • engaging in unsafe and extreme sex in exchange for more money; • fear of contracting COVID-19 while working but also fear of dying from hunger.
Singh & Kaur (2022, India) [71]	Qualitative research/ Face-to-face interviews/Thematic analysis	May 2020	To analyse the lived experiences of women in informal employment during the COVID-19 crisis	21 mixed occupations, 17–56 years	• severe economic stresses and income losses; • low caste women seen as carriers of the virus; • lack of income support from the government; • female-headed households disproportionately affected by economic constraints; • heightened food insecurity worse for women who often prioritised other family members; • absence of safe and reliable public transportation heightened social exclusion; • poor access to sexual and reproductive health services; • social ostracisation and religious-based discrimination.
Sithole (2022, South Africa) [72]	Qualitative research/ Face-to-face interviews and focus groups/Thematic analysis	June – July 2022	To assess the coping strategies utilised by female street traders during the COVID-19 pandemic in the City of uMhlathuze, KwaZulu-Natal	43 street traders, Black, 9 were undocumented migrants from Mozambique, majority between 31–40 years old,	• restrictions on street trading impacted ability to earn income; • inadequate infrastructure and lack of access to personal protective equipment limiting ability to earn and affecting health; • harassment by officials including confiscation of goods and payment of bribes; • affected differently based on, among others, nationality, age, and form or trade; • undocumented migrants unable to apply for trading permits and access grants or relief measures in place; • coping strategies include diversifying trading activities and shifting to mobile street traders; • importance of solidarity and social networks in coping.
Sumalatha et al. (2021, India) [73]	Mixed methods research/Telephone interviews/Manual analysis	Not specified	To assess the situation of domestic workers in relation to changed working conditions, livelihood and family dynamics during the pandemic and to document the state support systems in place to address the issues	12 domestic workers, majority between 42–49 years old	• unexpected employment and income shortage; • aggravation of gender-based discrimination; • increase of domestic violence; • hunger and food insecurity; • lack of food resulting in malnutrition and anaemia; • borrowing money from predatory money lenders to afford expenses; • disrupted health care access due to financial constraint; • increased burden of care; • deterioration in quality of life due to lack of time for rest and recreation.
Tshivhase & Moyo (2023, Zimbabwe) [74]	Qualitative research/ Face-to-face interviews/ Colaizzi’s method of data analysis	December 2020 – March 2021	To explore and describe the innovative approaches that were adopted to enhance HIV service delivery among female sex workers during the COVID-19 pandemic	10 sex workers, users of HIV prevention and treatment services, 31–45 years old	• received in-person support at home or in the community through outreach services; • multi-month dispensing of antiretroviral and prophylaxis medicines was convenient; • utilisation of telehealth in treatment management; • received telephonic support from sex-worker organisations in the form of counselling; • peers, non-organisational institutions, and families providing, money, and emotional support helped adherence to HIV treatment.
Vyas et al. (2023, India) [75]	Mixed methods research/Face-to-face interviews/Not specified	Not specified	To examine the effects of the pandemic on women domestic workers and street vendors, and to identify the variables that aided them in coping	50 street vendors and domestic workers, 18–49 years old	• loss of work and income; • difficulty finding alternative employment opportunities; • limited public transportation; • forced to borrow from informal sources such as money lenders at high interest rates; • acute food insecurity and reduced food consumption; • lack of ration card limited access to free food distribution; • increased burden of unpaid care work; • limited access to sexual and reproductive health services; • intimate partner violence associated with economic hardship and confinement in private spaces; • high levels of anxiety and physical manifestations of poor mental health; • street vendors - police harassment in the form of demands for bribes, verbal abuse, and physical violence; • domestic worker - reduction in negotiation powers, intensification of chores, and lower salaries; • women who had access to welfare measures by government and civil society organisations fared better • benevolence of employers critical in helping domestic workers.
Wandera et al. (2023, Kenya) [76]	Mixed methods research/Interviews and focus groups/Not specified	Not specified	To examines the social and economic impact of COVID-19 on women in the informal urban economy in the food and trade services sub-sectors	≥ 5 women working in food and trading services (exact number unspecified), 18–70 years old	• traditional expectation to shoulder a higher burden of home care; • financial concerns led women to send family members away from household; • unable to benefit from government relief mechanisms due to unfair registration process; • received support from non-governmental bodies; • difficulty accessing sexual and reproductive health services; • limited access to gender-based violence care services.
Wandera et al. (2023, Uganda) [77]	Mixed methods research/Interviews and focus groups/Not specified	Not specified	To understand the socioeconomic impact of the COVID-19 crisis and policy response on women in informal economy across a range of occupations in the services sub-sector	≥ 5 women working in food and trading services (exact number unspecified), 19–65 years old	• increased burden of domestic work; • food insecurity due to loss of income;• • women with disabilities being dependent on public transportation faced greater challenge to work; • cash transfer and food rations from government helped but distribution was limited; • use of digital platforms to conduct business transactions; • borrowed from money lenders; • limited access to sexual and reproductive health services due to permit requirement and transport costs; • economic hardship aggravated causes of gender-based violence; • physical beating and mistreatment by government security personnel.
Wasima & Rahman (2022, Bangladesh) [78]	Qualitative research/ Face-to-face interviews/ Qualitative content analysis	Not specified	To explore the economic vulnerabilities of housemaids amidst the pandemic	9 domestic workers, 28–48 years old	• loss of employment; • excess workloads; • inability to pay rent and bills; • food shortage; • inaccessible and inadequate government relief.
Wrigley-Asante & Frimpong (2024, Ghana) [79]	Mixed methods research/Face-to-face interviews/Thematic analysis	January 2022	To demonstrate how the livelihoods of Ghanaian women engaged in informal cross-border trading have been impacted by the COVID-19 pandemic and how they cope with the effects	20 cross-border traders, majority between 41–50 years old	• closure of borders, lockdown and a decline in economic activities resulted in income drop; • feelings of sadness and fear for the unknown; • turned to smuggling goods to overcome economic hardship; • risks of being arrested, attacked by armed robbers, accidents and other health challenges for using illegal routes; • using digital tools to connect with social networks and support livelihood.
Yadav & Jacob (2023, India) [80]	Qualitative research/ Telephone interviews/Not specified	June 2020	To analyse the socio-economic conditions, the impact of COVID-19 on women domestic workers and recommend strategies to uplift their lives	5 domestic workers	• abrupt loss of work and income; • poor hygiene practice and social distancing from living in slums; • food insecurity; • access to health facilities impacted due to lack of public transport; • insufficient financial relief and poor quality ration from the government; • those without identification document were disadvantaged in being rehired.
Zulfiqar (2022, Pakistan) [81]	Qualitative research/ Telephone interviews and face-to-face focus groups/ Inductive and deductive approach	July – October 2020	To examine the effect of COVID-19 pandemic and the disruption of global production networks on the lives of women homeworkers	52 home-based workers	• sudden loss of work and income; • increased exploitation as work increased but remuneration was poorer than before; • food insecurity; • inability to pay rent and utility bills; • increase of domestic violence; • some believed that COVID-19 was a hoax; • fear of contracting the virus was much less worrisome than hunger; • survival strategies include drawing from personal savings and taking in debt; • unable to get government aid hence relied on family and neighbours.

A third of all included studies were concerned with the experiences of sex workers (*n* = 18) [29, 37, 38, 41, 43, 46, 47, 49–51, 54, 55, 57, 59, 61, 66, 70, 74]. Others explored experiences of women in mixed informal occupations (*n* = 14) [30, 34–36, 48, 60, 62, 64, 67, 68, 71, 75–77], as domestic workers (*n* = 7) [39, 42, 53, 69, 73, 78, 80], traders/vendors (*n* = 5) [32, 44, 56, 72, 79], entrepreneurs (*n* = 3) [52, 58, 63], home-based workers (*n* = 2) [31, 81], labourers (*n* = 2) [40, 45], fisherwomen (*n* = 1) [65], and porter (*n* = 1) [33]. Fifteen studies included the experiences of women who were also migrants [29, 34, 36, 39, 46, 47, 49, 54, 56, 58, 64, 68, 72, 75, 80].

### Quality assessment

**Fig. 2:**
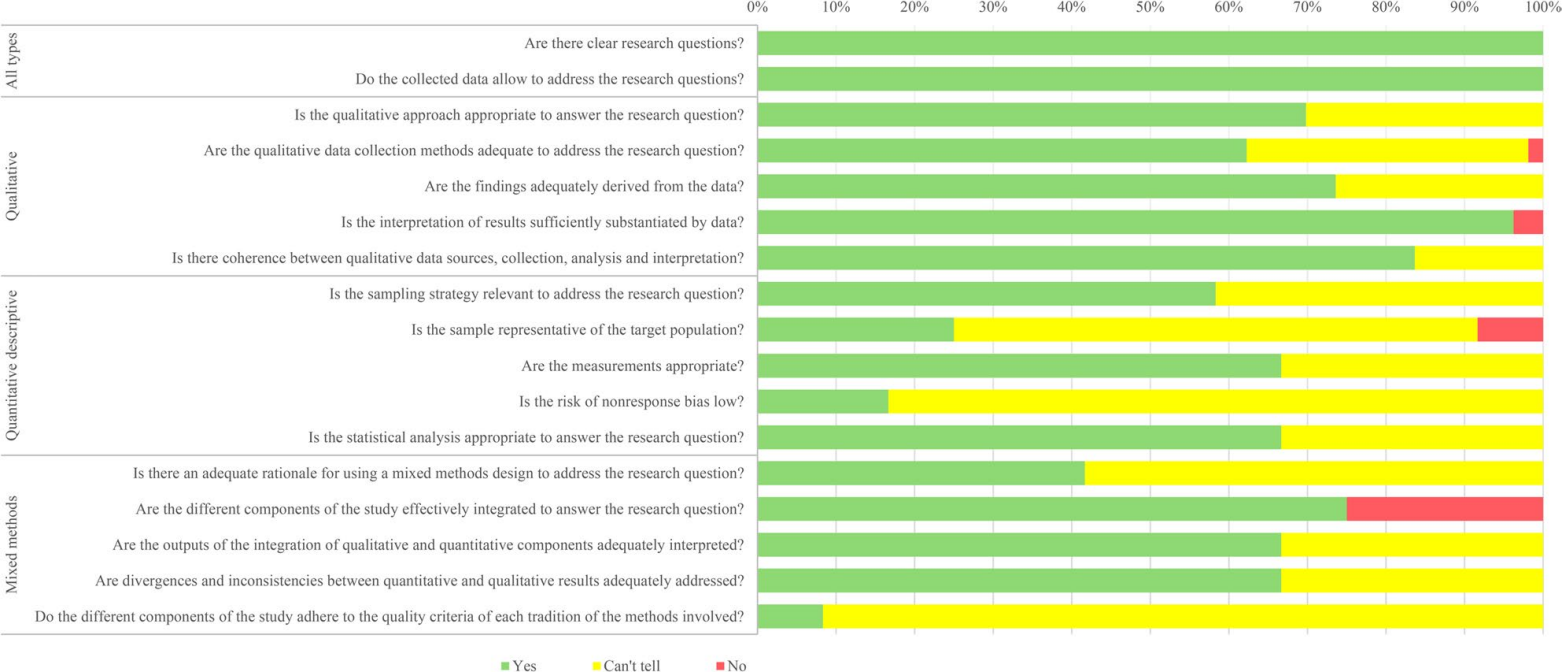
Assessment of the quality of the studies based on the Mixed Methods Appraisal Tool (MMAT)

The outcomes of quality assessment are presented in Fig. 2 and are available in full in Supplementary pp. 13–17. Overall, most of the included studies were of good quality. Seventeen qualitative studies [29, 34, 40, 41, 43, 46, 48, 50–52, 54, 57, 59, 60, 63, 69, 74] and one mixed methods study [38] fulfilled all assessment criteria. Three mixed methods studies only reported qualitative findings [30, 42, 49]. Common issues with qualitative studies were lack of clarity about appropriateness of approach and insufficient information about data collection methods. Common issues across mixed methods studies were inadequate reporting of design rationale, response bias and sample representativeness, and not meeting criteria for both qualitative and quantitative components.

### Themes

Thematic synthesis of included studies generated four themes central to the health and wellbeing experiences of women during the recent COVID-19 pandemic: (1) exacerbation of existing vulnerabilities; (2) negotiation of risks and resilience; (3) interconnectedness of health and wellbeing stressors; and (4) variable experiences across social locations. Studies contributing to the themes are found in Supplementary pp. 18–20. We present several quotations from the contributing studies to illustrate and support the themes and subthemes generated, with additional relevant quotations provided in Supplementary pp. 21–25.

#### Exacerbation of existing vulnerabilities

This theme was generated based on findings reported in 51 of the included studies. It encompasses factors which illustrate the cultural and socioeconomic context of women, generating and reinforcing their vulnerable position within society. These factors contribute to women’s poor access to resources and exert influences over women’s health and wellbeing during the pandemic.

##### Precarious work

With subsistence wages [37, 39, 40, 45, 60, 65, 68, 75, 81], limited employment rights, and social protection [35, 39, 40, 45, 60, 64, 69, 71, 73, 75, 78, 80], women’s work is precarious in nature. During the pandemic, women found themselves being made redundant bereft of recourse [34, 36, 39, 40, 53, 69, 71, 73, 75, 78, 80]. Lockdowns rendered low patronage and difficulty for women to peddle their goods or services, thus a downfall of their small operations [29–33, 35, 37, 38, 41, 43, 45–49, 51, 52, 54, 56–61, 63–66, 68, 70–72, 75, 77, 79, 81]. The pandemic took a sudden devastating toll on the already fragile livelihoods of women [29–41, 43–61, 63–73, 75, 77–81].

*“I worked as a housemaid in three different households. Since the government announced the lockdown*,* I have been informed by the families not to come for work. Initially*,* I thought that it might be for one or two days but as the days passed*,* the period became longer. Hence*,* I was worried about our livelihood because I had no income throughout the lockdown period.”* [53].

##### Household poverty

Women typically come from poor background [31, 33] and their household members typically also engaged in a variety of precarious employment [29, 31, 33, 35, 36, 53, 63, 71, 75, 78, 81]. Household economic situation was adversely affected following sudden loss of earnings particularly as they cannot benefit from unemployment protection [29, 31, 33, 35, 36, 53, 63, 71, 73, 75, 78, 81]. With little to no safety nets, women and their families were propelled deeper into poverty [31, 33, 36, 57, 60].

*“My son also worked on daily wages and helped me to maintain household expenses. The pandemic badly affected my work as I did not get more orders. My son also lost his wage work. We had small gold earrings and nose pins. We (mother-in-law*,* daughter-in-law and I) sold them to deal with the crisis.”* [31].

##### Patriarchal society

Perceived as menial, domestic chores such as cooking, cleaning, and caregiving were often disproportionately assumed by women. This workload amplified during the pandemic [34–36, 39, 42, 45, 48, 52, 56, 57, 60, 67, 69, 73, 75–78], limiting their productivity and preventing them from fully engaging in monetised work [31, 33, 35, 44, 52, 65, 67, 72, 75]. Women not only shouldered the responsibility of childcare and homeschooling with nursery and school closure [35, 36, 42, 48, 52, 56, 60, 67, 75, 77, 78], but also tended to the elderly and ill relatives [34, 35, 48, 60, 69]. As resources were strained, women were often expected to prioritise the needs of others over their own [34–36, 42, 60, 67, 71, 73]. Gender bias was also felt through comparatively better treatment of men by the employers [42, 73] and male privileges at home [35, 36, 71].

*“It is not that difficult for men*,* but for women*,* it is really hard. You know*,* you are responsible with the household chores*,* and then there is the fear of being infected. Your thinking is completely altered. And on the top of that*,* there are the kids and the husband*,* who are constantly asking for food.*” [69].

Compared to their men counterparts, women found it more difficult to find work again particularly with the restrictions imposed [31, 40, 65, 75, 81]. Their loss of income, and so of economic productivity, translated into a reduced ability to exercise voice and agency for women [34, 39, 42, 79]. The pandemic accentuated a social hierarchy based on male dominance, rendering women into submissive and dependent roles [29, 47, 63].

*“Earning money gives us confidence*,* we get respect from our husband and in-laws as we bring in food. The pandemic took away that sense…. the ability to move outside the house freely and make decisions independently.”* [34].

##### Stigma and discrimination

Some work performed by women tend to be perceived as inferior, dirty or illegal [29, 37, 39–42, 49–51, 61]. COVID-19 brought new form of stigma, with women demeaned as carriers of virus [31, 34, 37, 39, 41, 42, 55, 66, 71, 75, 78] due to being poor [34, 71] and from lower class or caste [39, 71]. Some were stereotyped as reservoirs of infection because they worked as sex workers [41, 55], lived in slums [78] or transgressed pandemic protocols [37, 42, 66]. Pandemic heightened the othering of women [34, 36, 39–41, 49, 50, 56, 59] and regulations were used to excuse the derogatory and discriminatory treatment of them [29, 31, 34, 39, 42, 55, 71, 78].

*“I lost my stable work during the first wave of Covid as my employer thought I might carry the virus from the slum.”* [78].

#### Negotiation of risks and resilience

This theme was generated based on the findings of all 53 included studies. It pertains to how women perceived, felt, and behaved towards COVID-19 pandemic. Predisposed by their existing vulnerabilities, women faced an increased risk of contracting the virus as well as the debilitating social consequences of both infection and its associated control measures. Women’s attitudes and actions reflect their dire need for sustenance, but also indicates high level of adaptability and resilience.

##### Mixed patterns of belief and protective behaviours

Women utilised mass media and social media for pandemic-related information [32, 35, 36, 54, 57, 62, 64], although awareness was poor at outset [31, 33, 42, 64]. Fear related to perceived risk of being infected and consequences of infection was prevalent [31, 36, 37, 39, 42, 46–48, 54, 55, 57, 59, 69, 76, 78]. Scepticism and conspiratorial ideation about COVID-19 were also expressed, indicating mistrust of governments [47, 54, 81]. Some revised their beliefs however after experiencing the disease or witnessing others’ experiences [54].

*“There is no COVID. This is Imran Khan’s [the Prime Minister’s] concoction.”* [81].

Women generally possessed knowledge, although not always optimal, about measures to tackle COVID-19 spread and tried to conform [29, 32, 36–39, 45–47, 49, 54, 55, 57, 65, 69, 70]. Their responses were grounded in the understanding that measures were necessary for the wellbeing of themselves and others [29, 36–38, 45, 46, 49, 55]. Another reason for compliance was fear of arrest or fines for breaching regulations [46, 47, 58, 65]. Where women failed to observe pandemic regulations such as curfew or mask-wearing, economic need, limited resources, and impracticability of the practices were often cited [29, 34, 36–39, 44, 45, 47, 48, 50, 52, 54, 58, 60, 61, 65, 66, 70, 72, 80].

Dissonant views and responses to pandemic measures could also be attributed to fatalism and preordination [43, 62]. Attitudes towards COVID-19 vaccine additionally were mediated by confidence in its safety and effectiveness [54]. On the other hand, vaccine hesitancy and refusal related to misinformation about its effects such as infertility and death had been observed [42, 54, 57, 62]. Vaccination decision in some were tied to the threat of termination by their employers and state-imposed sanctions on unvaccinated individuals [62].

*“We are confused whether we should take vaccination; because it will affect our fertility.”* [42].

##### Risk and effect of infection

Women were at high risk of being exposed to and contracting COVID-19 because their work often involved being in public spaces or in close proximity to others [34, 36, 39, 52, 60, 67, 72]. Sex workers were particularly susceptible as their work was not conducive to wearing personal protective equipment [38, 41, 46, 47, 50, 54]. Besides poor consideration of social distancing protocol by employers [42, 45, 73, 78], infection also loomed large for women due to overcrowding and unsanitary living conditions [36, 44, 46, 60, 80].

*“There are some precautions that don’t align with the nature of my work. For instance*,* wearing face masks*,* or washing hands regularly during sex is not possible and this is why I feel at risk.”* [38].

Despite experiencing symptoms, some were unaware of their infection status as testing was costly [54]. COVID-19 not only afflicted women with physical illness [52, 54, 69], but it also left them vulnerable to dismissal and ostracisation, so some tried to suppress signs of infection [39, 71, 78]. Reports of women being coerced by employers to continue working and denied medical assistance had been raised [46].

##### Exposure to harm

No choice but to prioritise their economic needs over health concerns, women worked clandestinely to circumvent lockdowns and surveillance [34, 45–47, 51, 54, 61, 65, 66, 75]. This exposed them to dangers arising from the risk taken and the need to obscure themselves [33, 45, 46, 65, 79], as well as blackmail threats [46]. Women caught breaking law faced hefty fine, arrest, extortion and even brutality from law enforcement [29, 37, 39, 41, 42, 44, 51, 55–57, 59–61, 64, 65, 72, 75, 77, 79, 81]. Many had to capitulate to avoid further harm [29, 59, 75].

Given their low bargaining power, women were unable to refuse poor employment terms they previously eschewed [39, 40, 56, 73, 75]. Many were taken advantage of by middlemen or employers who avoided paying commensurate prices or wages [31, 39, 40, 42, 45, 60, 71, 73, 75, 78, 79, 81]. To survive financially, women were forced to borrow from informal channels including predatory lenders, with many exploited with unjust loan terms and extortionate interest rates [36, 37, 45, 47, 53, 59, 67–69, 73, 75, 77, 78, 81].

Furthermore, bar and brothel closures pushed sex workers to solicit on the streets where they were prone to street crime and harassment [29, 54]. Some were lured to meet clients in unvetted spaces which posed safety risks [37, 49, 66, 70]. Online sex work, which was an alternative way of earning for some, also put women at peril of having their intimate information abused or payment cancelled [37, 47, 49, 61, 66]. The desperation for money compelled them to accept any clients, including those who demanded unsafe sex and paid less [29, 37, 38, 41, 46, 47, 51, 55, 57, 59, 66].

*“There is much more danger there. Men on the street are more abusive. At least one man took out a gun on me*,* because he wanted me to give him ass [anal sex]. It is much more difficult than working in a business. In a business*,* they take care of you*,* even the waiters themselves notice who you are going with.”* [54].

##### Enabling factors

Having multiple income streams [48, 51, 56, 75, 81] and welfare support from their governments [31, 45, 53, 56, 61, 69, 72, 77] as well as dissaving and sale of assets [29, 31, 37, 41–43, 45, 49, 53, 58, 63, 69, 71, 73, 75, 77, 78, 81] provided relief somewhat for women and their households. While some were able to avail in-demand skillsets such as sewing face masks [30, 43, 48, 52], many diversified their work practices by adopting smartphones and online platforms for virtual transactions, advertisement, and communication with customers [29, 31, 35, 38, 41, 47–49, 52, 59, 61, 64, 66, 67, 73, 75, 77]. Social media was utilised to connect with loved ones [32, 35, 64, 74] and to mobilise donations from philanthropists [48, 56, 64]. Women also relied on social media, in addition to broadcast media, as important sources of pandemic-related information [32, 64]. Moreover, the use of digital technologies allowed women to conveniently access telehealth services and treatment during lockdowns [55, 74].

Given limited personal capacity and state protection, a critical resource for women was the collective support afforded by their interpersonal, community and organisational networks [29–31, 34, 36, 37, 43–48, 50, 53, 55, 56, 61, 63–65, 67, 70–72, 74–77, 79, 81]. Women relied on families and friends for various forms of instrumental assistance and emotional comfort [29–31, 34, 36, 37, 43–48, 50, 53, 55, 63–65, 67, 70–72, 74, 75, 77, 79, 81]. Online communities [48, 52, 64, 74], charitable organisations [29, 36, 43, 45, 47, 49, 50, 53, 55, 61, 65, 67, 70, 71, 74–76, 81], and places of worship [50, 56, 71, 73] also stepped in to fill the void of government’s relief measures.

*“Some NGOs are mobile*,* they are always with us in difficult times*,* and some have offices in town. They helped us with food parcels and sanitary towels during the pandemic. It became clear that without them*,* our livelihoods would have been difficult as we would have never been able to cope with government regulations and lack of state support.”* [61].

Besides physical activities and self-care, women cultivated resilience and optimism to cope with their experiences [29–31, 33, 45, 48, 50, 54, 57, 68, 75]. Prayers and spiritual connection were also helpful during the stressful times [31, 35, 45, 48, 50, 54, 56, 78].

#### Interconnectedness of health and wellbeing stressors

This theme was generated based on findings reported in 52 of the included studies. It underscores how challenges in one domain of women’s health and wellbeing could spill over into challenges in several other facets of their lives, illustrating the interconnectedness of the situation. Financial strain was the primary stressor for women however. With job loss and subsequent pecuniary penalty, women experienced diminished ability to afford even basic needs including food, shelter, and healthcare. These stressors were not independent of one another, but rather, interact and gave rise to a synergistic effect on women’s health and wellbeing.

##### Financial strain

Losing livelihoods and struggling to find alternative work, income for women reduced drastically or in some cases ceased entirely. Barely eking out a living before, the pandemic magnified their financial vulnerability, particularly with limited savings and minimal aid to cushion the shock [29, 31–37, 39, 41, 43–50, 52–54, 56–58, 60–64, 66–68, 71–73, 75–78, 80, 81]. Some were unable to benefit from relief measures due to unmet eligibility requirements [36, 37, 44, 45, 48–50, 52–54, 56, 58, 60, 61, 72, 75, 81] while others were excluded because of limited awareness and difficulties navigating the application process [36, 45, 46, 53, 60, 72, 75, 78]. Impoverished, women struggled to meet their basic needs and those of their families without compromising their health and wellbeing or essential livelihood assets [29–31, 33, 36, 38–42, 44, 46, 47, 49, 51, 53–61, 63, 65, 67, 69, 70, 73, 75, 78–81]. Many accumulated debt which further trapped them with financial insecurity [36, 37, 45, 47, 53, 59, 67–69, 73, 75, 77, 78, 81].

*“We are going through a bad time. We become jobless when the lockdown is declared. But people were saying we will get support from the government. They have not done anything for us. We do not have a ration card like most people living here. I applied for the ration by filling an online form. Still*,* I did not get ration or financial support. The case is the same for most of the people here…”* [36].

##### Physical and mental health

Insufficient rest from increased workload negatively affected women’s physical health [30, 35, 36, 42, 48, 73, 78]. To compensate for tightened budget, women forwent basic hygiene needs such as soap and menstrual materials [29, 36, 41, 58, 65].

Women experienced anxiety and depression furthermore, partly tied to the enormous pressure of making ends meet under pandemic constraints and uncertainties about the future [30, 33–37, 39, 41, 45–48, 50, 53, 54, 57, 59, 60, 63–65, 67–69, 71, 73, 75, 78, 79, 81]. Mothers especially felt inadequate for not being able provide for their children [33, 43–45, 48, 50, 63, 64, 79, 80]. The deterioration in mental health could also be explained by social isolation instigated by COVID-19 [30, 34–37, 39, 45–47, 57, 59, 64, 69, 73, 75]. The high level of mental stress experienced by women manifested in psychosomatic symptoms including headaches, fatigue, and sleeplessness [37, 42, 46, 50, 69, 75, 79].

*“I didn’t do anything. I just kept sitting. I had tension about how the expenses of the house would go and how I would pay the rent of the room. I just kept thinking this and sometimes I thought I should die. The situation in my house had deteriorated a lot. Whenever I remember that time*,* I still cry.”* [75].

##### Shelter and food security

One of the foremost issues resulting from financial predicament was housing insecurity. Women were threatened with eviction and coerced to leave homes as they were unable to pay rent [29, 36, 37, 39, 41, 46, 53–56, 59, 63, 64, 69, 70, 75, 78, 81]. Those sheltering in family homes meanwhile endured abuse by their relatives to avoid being thrown out [33, 39, 46, 80].

*“In May 2020*,* when I lost my work*,* I told my landlord that I would pay the monthly rent in a few days. You know what they did? They shut me out for seven days…People do not care whether you lost your employment or not*,* they need their payments on time.”* [78].

Buying and stocking food were significantly affected [29, 31, 34, 36–39, 41, 42, 44, 46, 49, 51, 53–57, 59, 60, 63, 65–68, 70, 71, 73, 75, 77–81], especially with inflation [29, 36, 54, 56, 75, 81], short supplies [36, 75, 79], and limited opening hours of shops [53, 71]. In many cases, women sacrificed their own meals to afford other essential needs [31, 36, 37, 45, 53, 54, 59, 81] or to shield their families [29, 42, 51, 67, 71, 73, 75, 78]. This led to poorer physical health, with symptoms of anaemia and nutritional deficiencies being described [42, 55, 71, 73, 75, 80]. In addition, women reported poor adherence to medicines to avoid taking them on empty stomachs [37].

##### Interpersonal tension

Women’s familial relationships worsened, with economic stress attributed as key aggravating factor [33, 37, 39, 40, 42, 50, 53, 57, 73, 75, 77, 81]. Stress from being confined and increased care burden also intensified household conflicts during the lockdown period [42, 50, 53, 67, 69, 70, 73, 78]. This subsequently provoked abusive behaviours towards women and a surge in domestic violence [33–35, 37, 39, 40, 42, 46, 47, 53, 57, 67, 70, 73, 75, 77, 78, 81]. At work, besides disagreement over attitudes towards protective measures [44], women witnessed reduced solidarity among themselves following fierce market competition brought by economic decline [29, 49, 50, 56, 58].

*“There was high stress and my husband would get angry on small issues. He would constantly abuse me and say that we are cursed. Earlier (before the lockdown)*,* we would be busy working the whole day and would see each other only at night…. we would hardly get time to talk*,* leave alone fight….The fights and the abuses increased after the lockdown.”* [34].

##### Barriers to healthcare

With COVID-19 dominating public agendas, many resources were diverted to respond to the exigencies [42, 55, 57, 71, 75]. Healthcare needs of women were hampered due to poor availability of services [33, 36, 37, 49, 53, 54, 57, 66, 76, 80], ineligibility to access [36, 49, 54, 64], limited operations [36, 53, 55, 57, 75, 76], disruption of supplies [55, 66], movement restrictions [37, 42, 55, 57, 71, 75–77], and suspension of public transport [57, 71, 75, 77, 80] during the crisis.

*“It was hard for us to go to the hospital for delivery. We had curfew*,* women were in labour and could not go to the hospital. The curfew was from 7 pm to 6 am in the morning… the first challenge was getting to the hospital because we had curfew.”* [76].

Financial struggle remained a lament for many [36, 37, 39, 42, 45, 49, 53–55, 57, 63, 66, 73, 75, 76, 80, 81], but women also avoided seeking treatment due to fear of contracting COVID-19 [55, 57, 76] and stigma in healthcare settings [49, 55]. These issues led to neglect of non-emergency care including for chronic conditions and sexual and reproductive health [42, 55, 57, 66, 71, 75–77].

##### Health behaviour

As a way of escaping pandemic stress, women increased their alcohol and substance use [49, 50, 66]. However, fall in disposable income have also compelled lower consumption amongst women [37, 41]. Those wanting to quit addiction reported suffering from withdrawal symptoms due to reduced access to counselling and detoxication services [41]. The difficulty in accessing healthcare services further led to women self-medicating with herbal medicines or home remedies [54, 57, 75]. For sex workers moreover, desperation for money enticed them into high-risk sexual behaviours despite awareness of the harm [41, 55, 59, 66, 70].

*“Some clients request us to make oral sex while some others ask to have sex without condom. I know very well that these sexual practices can expose us for different kinds of sexually transmitted diseases including HIV/AIDS. I had previously refused such kinds of sexual requests. After the outbreak of the corona pandemic*,* I become careless to protect myself from such kinds of unsafe sexual intercourses. You know why… I would lose my clients if I did not fulfil their sexual needs.”* [41].

#### Variable experiences across social locations

This theme was generated based on findings reported in 45 of the included studies. It recognises that women are far from a homogenous group and considers diverse experiences. The health and wellbeing experiences of women were shaped not only by informality and gender roles, but also other critical factors that intersect such as migration status, occupation type, age, race, ethnicity, class, caste, religion, and ability/disability. Women’s unique mix of social identities within interlocking systems of power determine how the pandemic had affected them and how they were able to respond.

##### Sex workers

Beyond common experiences shared with women in other types of informal work, sex workers tackled an additional layer of vulnerability. Even amongst themselves, there is a hierarchal difference based on factors such as race, migration status, work venue, and the socioeconomic status of their clientele [29, 49, 51, 54]. Sex workers were particularly targeted owing to connotations of criminality and immorality attached to their work [29, 37, 41, 51, 59]. They reported being discriminated against by healthcare providers and excluded from relief measures because of their occupation. Often they felt the need to conceal their identity, and having to disclose this information at checkpoints or on aid applications compromised their privacy and caused them severe distress [29, 50, 54, 59, 61, 70]. Additionally, engagement in unprotected sex in order to be paid more put them highly exposed to sexually transmitted infections [41, 55, 59, 66].

*“But if you’re a sex worker you’re really not eligible even with a legitimate business because they’ve got a clause in there that anything to do with adult entertainment or the industry or things like that are not eligible for these funds.”* [49].

##### Migrant women

Migrant women sustained further challenges because of the framing of them as outsiders. Irregular, non-citizen or non-resident status excluded some from health services and relief measures reserved only for those eligible [36, 46, 47, 49, 54, 56, 64, 68, 72, 75]. Some dreaded being made illegal as lockdown prevented in-time renewal of visas or permits [64]. Not only did they struggle to find work [72, 80], those without identification document also feared arrest and deportation following tightening of surveillance during the pandemic [29, 47, 56, 64]. Increased xenophobic threats amid the crisis augmented their plight [56, 58, 64].

*“The manager told us that we shouldn’t go [for health check] because they would ask us how we had become infected and they would ask us for our papers.”* [46].

Without nearby family support, migrant women relied on solidarity network of others coming from the same place as them [34, 56]. Those moving back to their villages endured arduous journey along the way or were stuck due to lack of transport [34, 36, 39]. For some, returning to their villages after years away brought a loss of a sense of purpose and belonging [34].

##### Identity categories

Despite being afflicted by similar pandemic policies, women’s experiences manifested differently based on their social identities. In India for example, Muslim women experienced harassment and boycott after a Muslim congregation was blamed for an outbreak [39, 71]. By the same token, women of colour or from low caste faced heightened sense of otherness and discriminatory practices that limited their economic opportunities [33, 34, 40, 49, 71]. Older and disabled women were more vulnerable to severe COVID-19 illness and in restrictive pandemic environments [29, 37, 56, 65, 72, 77].

*“We run a grocery shop in the village*,* and were supplying rations through home delivery during the lockdown. After my brother-in-law went out for some work for two days*,* he was branded a Tabhligi and our entire family was quarantined for 3 weeks…We had already stocked up rations for home deliveries when we were quarantined. We suffered heavy losses. We have been treated like criminals.”* [71].

Differences in women’s experiences were also mediated by the sector and scale of trades conducted. Comparably, women with smaller, less stable trades and those dealing in non-essential or perishable items sustained more punishing consequences [44, 52, 72, 75, 77]. Working in public spaces, traders/vendors and street-based sex workers were more susceptible to experiencing aggression from law enforcers given their more direct and regular encounters [29, 37, 41, 44, 51, 56, 58, 59, 61, 62, 72, 75, 77]. As breadwinners and chief caregivers, distinct struggles of women who were single mothers were accentuated by the pandemic as they fended for their households through school and childcare closures [33, 37, 45, 46, 51–53, 55, 56, 60, 63, 65, 71, 75, 77, 79].

##### Positive experiences

Women capitalised on business opportunities emerging during the pandemic, profiting from high-demand items such as masks and grocery delivery [30, 48, 52, 65, 77]. Lockdown also afforded more time with family at home [30, 43, 48]. Those with older children benefitted from assistance with work and house chores, which subsequently allowed women more rest [30, 56, 81]. Additionally, women found the provision of outreach health services by governments or civil society organisations convenient and supportive of their needs [37, 74, 76, 77].

*“Later*,* I realised that because of border closures*,* there were shortages of nose masks*,* so I seized the opportunity and began to sew nose masks made from the fabrics I work with. In no time*,* I was really making some good money…so I really did not feel much financial difficulties.”* [30].

## Discussion

This review provides insight into a constellation of variables that shaped health and wellbeing experiences of women informal workers during the recent pandemic. A prominent feature of this review lies in shifting the focus from direct health consequences of COVID-19 to the underlying socioeconomic stressors, particularly financial insecurity, that shaped women’s lived experiences. Our findings highlight how abrupt loss of income, as pandemic restrictions eliminated the means of making a living, triggered a cascade of challenges in many spheres of daily life for women. As underscored in a comprehensive review examining the correlation between COVID-19 and health inequalities [82], financial insecurity influenced exposure, access and outcomes during the pandemic, revealing that many of the impacts were rooted not in health conditions, but in long-standing social disadvantages. We echo multiple prior studies exploring the impact of COVID-19 on vulnerable sections of society [15, 83, 84], reiterating the prominence of financial strain and its knock-on effects across multiple domains of health and wellbeing.

A key contribution of this review is its illustration of the interconnected nature of stressors. Rather than operating in isolation, challenges such as food insecurity, housing instability and familial tension clustered among disadvantaged women, revealing the limitations of viewing health determinants as discrete factors. While existing models of health determinants commonly conceptualise health outcomes as the cumulative result of individual factors [25, 85], our findings advance this discourse by characterising women’s experiences as a complex product of multifactorial and interrelated pathways. We also posit pandemic response policies as a structural determinant which influenced not only the direct health repercussions of COVID-19 but also indirect outcomes such as income and healthcare access. Although governments implemented measures to contain viral transmission, many failed to ensure adequate social safety nets to support those most affected. The experiences captured in this review reflect a broader pattern of neglect and are in line with a comparative analysis of pandemic policies across 15 countries [86] which highlight the inadequacy of protective measures to address the needs of vulnerable populations. We therefore stress the need for an equity-centred policy framework with multidisciplinary, cross-silo approaches to better anticipate, prevent and address arising issues in future pandemics [87, 88].

**Fig. 3:**
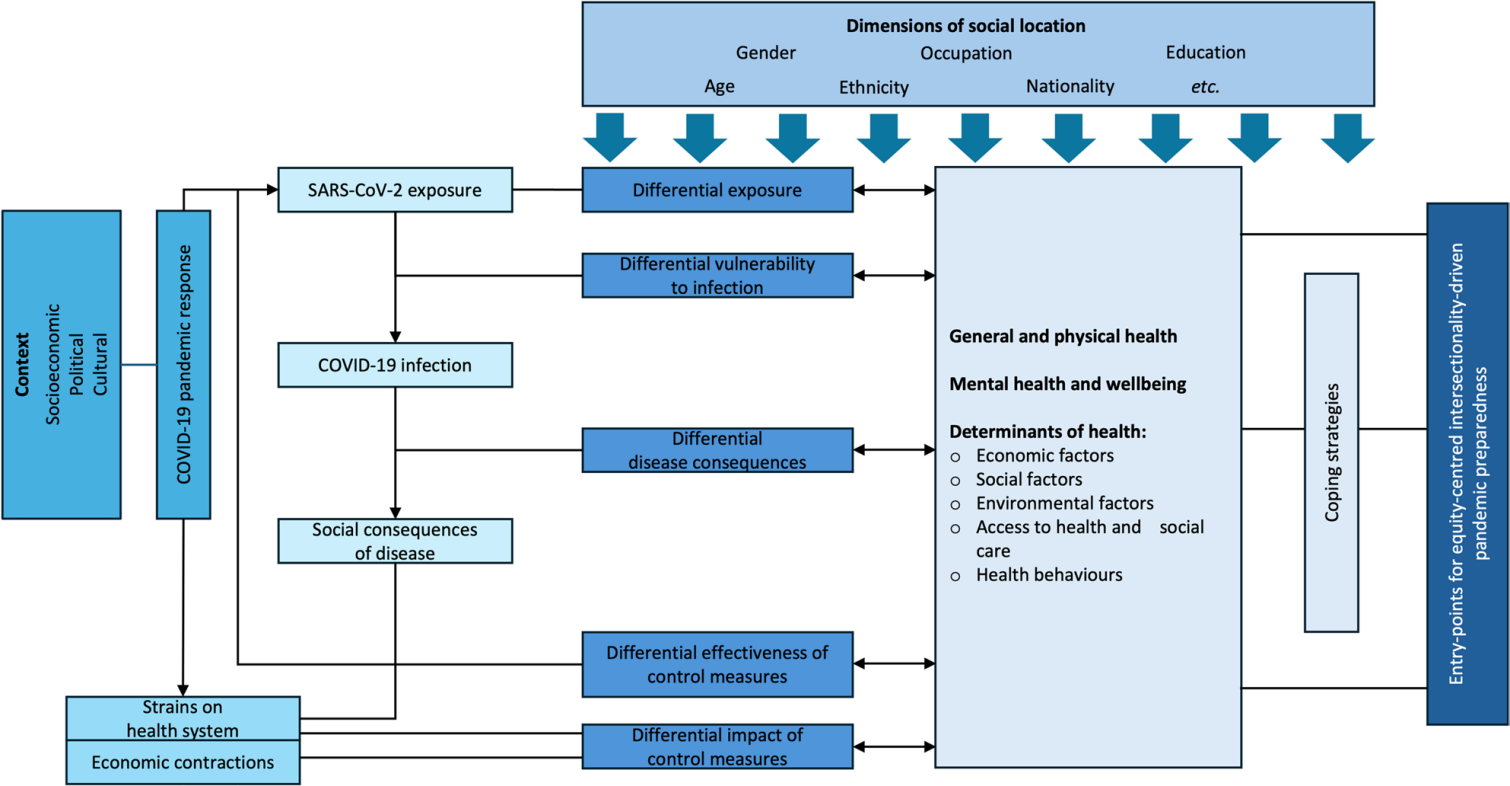
A conceptual mapping of the health and wellbeing experiences of women informal workers during the COVID-19 pandemic

Our review resonates with findings from a scoping review of sex workers’ experiences during the pandemic by Brooks, Patel, and Greenberg [15], which similarly highlighted how limited institutional support heightened vulnerability and compromised access to essential services. Their call for intersectional approaches is directly addressed in our review – recognising that women informal workers are a heterogenous group, we account for the interaction of multiple social identities such as occupational characteristics, migration status, ethnoracial, class/caste, and religion in our synthesis of their experiences. Impact endured by women was linked to their position within the hierarchies, with those at the intersections of multiple forms of disadvantages likely worse off. We present a conceptual model (Fig. 3) that maps the pathways through which the health and wellbeing of women informal workers were impacted during the COVID-19 pandemic, depicting the multifaceted and intersectional nature of their experiences. By situating women’s pandemic experiences within broader systems of social stratification and foregrounding their multi-layered experiences, our review enriches a growing body of work that calls for context-sensitive, intersectionality-informed approaches to pandemic preparedness [89–91], with the aim of ensuring that the most marginalised voices are not only heard but meaningfully addressed in policy and practice.

### Strengths and limitations

To the best of our knowledge, this is the first systematic review of qualitative evidence synthesising the health and wellbeing experiences of women informal workers during the COVID-19 pandemic. The qualitative emphasis of this review reveals critical experiential insights that may not be readily apparent through quantitative analysis alone. We also presented snapshots on the capacities and resources of women rather than solely focusing on their risks and vulnerabilities, thus fostering a strength-based approach to their health and wellbeing.

Despite endeavouring to include a diverse range of sources, certain limitations of this review should be acknowledged. Although we have made efforts to include grey literature such as dissertations, theses and reports from relevant organisations, predominance of published literature may introduce a degree of publication bias, potentially constraining the diversity of perspectives represented in the review. Additionally, exclusion of non-English studies may have further narrowed the breadth of women’s experiences considered, possibly overlooking culturally-specific insights that would have enriched our findings.

Majority of included studies were conducted in low- and middle-income countries reflecting the prevalence of informality in these economies. As such, our review may have more relevance to settings with similar economies and social protection models. Albeit differences between countries in terms of severities of (and responses to) the pandemic, all countries indicated imposition of some form of mandatory isolation or social distancing measures to contain COVID-19 spread. The review also included studies from heterogenous occupational groups, geographical contexts, and methodological approaches, which presents challenges for direct comparison of findings. However, many of the issues explored were not unique to specific context. We discerned considerable overlap in women’s experiences across varied settings, suggesting that the core themes are broadly relevant despite contextual differences.

### Research implications

A deeper understanding of women’s experiences creates critical opportunities for policymakers and other stakeholders to tackle the specific needs of this oft-overlooked category of women workers, many of whom have been struggling even before the pandemic. Identifying deficit- and strength-based needs, several areas where sustainable improvements could be made include: (i) expansion of social protection system to informal workers; (ii) skill and technology training; (iii) provision of accessible childcare services that accommodate diverse employment situations; (iv) strengthening prevention and redress of violence against women; and (v) capacity building of women informal workers collectives. There is a need for further research to address the health and wellbeing of women beyond COVID-19 pandemic, its long-lasting impact and strategies for post-pandemic recovery. Moving beyond documenting the inequities experienced, focus should be on analysing policies that contributed to them and exploring ways of optimising outcomes in future pandemics.

## Conclusions

Bringing together evidence from 53 studies, our review captured a largely deplorable picture of the health and wellbeing of women informal workers who have been providing lifeline services to society. At the intersection of multiple vulnerabilities, they bore brunt not only from the interplay between pandemic restrictions and decent work deficits, but also from deep-rooted gendered norms and discriminatory practices. It is pivotal that we harness the momentum triggered by the recent pandemic to establish an enabling environment for women informal workers. Our review emphasises equity-centred pandemic preparedness effort, which must incorporate intersectionality-informed approach tailored for specific context.

## Supplementary Information

Supplementary Material 1.

## Data Availability

Data used in this review have been reported in the text, tables and figures. All other relevant data are included in the supplementary file.
