# Supplementary material for: Health and wellbeing experiences of women informal workers during the COVID-19 pandemic: a qualitative systematic review
Source: BMC Public Health. 2025 Nov 28;25:4356. doi: 10.1186/s12889-025-25728-z (PMC12751337; doi:10.1186/s12889-025-25728-z)
Supplement: Supplementary file 1 — Supplementary Material 1. [file 12889_2025_25728_MOESM1_ESM.docx]

Supplementary Material

**CONTENT OF SUPPLEMENTARY APPENDIX**

| A. | PRISMA 2020 checklist | p 3-4 |
| --- | --- | --- |
| B. | ENTREQ checklist | p 5 |
| C. | Search strategy for selected bibliographic databases | p 6-8 |
| D. | Excluded studies | p 9-12 |
| E. | Overview of quality assessment using Mixed Methods Appraisal Tool | p 13-17 |
| F. | Overview of themes contained in included studies | p 18-20 |
| G. | Relevant extracted quotations aligning with themes and subthemes | p 21-36 |

# A. Preferred Reporting Items for Systematic Reviews and Meta Analyses (PRISMA) 2020 checklist

| **Section and Topic** | **Item #** | **Checklist item** | **Location where item is located** |
| --- | --- | --- | --- |
| **TITLE** | | | |
| Title | 1 | Identify the report as a systematic review. | Manuscript: title |
| **ABSTRACT** | | | |
| Abstract | 2 | See the PRISMA 2020 for Abstracts checklist. | Manuscript: abstract |
| **INTRODUCTION** | | | |
| Rationale | 3 | Describe the rationale for the review in the context of existing knowledge. | Manuscript: introduction |
| Objectives | 4 | Provide an explicit statement of the objective(s) or question(s) the review addresses. | Manuscript: introduction |
| **METHODS** | | | |
| Eligibility criteria | 5 | Specify the inclusion and exclusion criteria for the review and how studies were grouped for the syntheses. | Manuscript: methods |
| Information sources | 6 | Specify all databases, registers, websites, organisations, reference lists and other sources searched or consulted to identify studies. Specify the date when each source was last searched or consulted. | Manuscript: methods |
| Search strategy | 7 | Present the full search strategies for all databases, registers and websites, including any filters and limits used. | Appendix C |
| Selection process | 8 | Specify the methods used to decide whether a study met the inclusion criteria of the review, including how many reviewers screened each record and each report retrieved, whether they worked independently, and if applicable, details of automation tools used in the process. | Manuscript: methods |
| Data collection process | 9 | Specify the methods used to collect data from reports, including how many reviewers collected data from each report, whether they worked independently, any processes for obtaining or confirming data from study investigators, and if applicable, details of automation tools used in the process. | Manuscript: methods |
| Data items | 10a | List and define all outcomes for which data were sought. Specify whether all results that were compatible with each outcome domain in each study were sought (e.g. for all measures, time points, analyses), and if not, the methods used to decide which results to collect. | Manuscript: methods |
|  | 10b | List and define all other variables for which data were sought (e.g. participant and intervention characteristics, funding sources). Describe any assumptions made about any missing or unclear information. | Manuscript: methods |
| Study risk of bias assessment | 11 | Specify the methods used to assess risk of bias in the included studies, including details of the tool(s) used, how many reviewers assessed each study and whether they worked independently, and if applicable, details of automation tools used in the process. | N/A |
| Effect measures | 12 | Specify for each outcome the effect measure(s) (e.g. risk ratio, mean difference) used in the synthesis or presentation of results. | N/A |
| Synthesis methods | 13a | Describe the processes used to decide which studies were eligible for each synthesis (e.g. tabulating the study intervention characteristics and comparing against the planned groups for each synthesis (item #5)). | Manuscript: methods |
|  | 13b | Describe any methods required to prepare the data for presentation or synthesis, such as handling of missing summary statistics, or data conversions. | Manuscript: methods |
|  | 13c | Describe any methods used to tabulate or visually display results of individual studies and syntheses. | Manuscript: methods |
|  | 13d | Describe any methods used to synthesize results and provide a rationale for the choice(s). If meta-analysis was performed, describe the model(s), method(s) to identify the presence and extent of statistical heterogeneity, and software package(s) used. | Manuscript: methods |
|  | 13e | Describe any methods used to explore possible causes of heterogeneity among study results (e.g. subgroup analysis, meta-regression). | N/A |
|  | 13f | Describe any sensitivity analyses conducted to assess robustness of the synthesized results. | N/A |
| Reporting bias assessment | 14 | Describe any methods used to assess risk of bias due to missing results in a synthesis (arising from reporting biases). | N/A |
| Certainty assessment | 15 | Describe any methods used to assess certainty (or confidence) in the body of evidence for an outcome. | Manuscript: methods |
| **RESULTS** | | | |
| Study selection | 16a | Describe the results of the search and selection process, from the number of records identified in the search to the number of studies included in the review, ideally using a flow diagram. | Manuscript: results |
|  | 16b | Cite studies that might appear to meet the inclusion criteria, but which were excluded, and explain why they were excluded. | Appendix D |
| Study characteristics | 17 | Cite each included study and present its characteristics. | Manuscript: results |
| Risk of bias in studies | 18 | Present assessments of risk of bias for each included study. | Manuscript: results, Appendix E |
| Results of individual studies | 19 | For all outcomes, present, for each study: (a) summary statistics for each group (where appropriate) and (b) an effect estimate and its precision (e.g. confidence/credible interval), ideally using structured tables or plots. | N/A |
| Results of syntheses | 20a | For each synthesis, briefly summarise the characteristics and risk of bias among contributing studies. | N/A |
|  | 20b | Present results of all statistical syntheses conducted. If meta-analysis was done, present for each the summary estimate and its precision (e.g. confidence/credible interval) and measures of statistical heterogeneity. If comparing groups, describe the direction of the effect. | N/A |
|  | 20c | Present results of all investigations of possible causes of heterogeneity among study results. | N/A |
|  | 20d | Present results of all sensitivity analyses conducted to assess the robustness of the synthesized results. | N/A |
| Reporting biases | 21 | Present assessments of risk of bias due to missing results (arising from reporting biases) for each synthesis assessed. | N/A |
| Certainty of evidence | 22 | Present assessments of certainty (or confidence) in the body of evidence for each outcome assessed. | N/A |
| **DISCUSSION** | | | |
| Discussion | 23a | Provide a general interpretation of the results in the context of other evidence. | Manuscript: discussion |
|  | 23b | Discuss any limitations of the evidence included in the review. | Manuscript: discussion |
|  | 23c | Discuss any limitations of the review processes used. | Manuscript: discussion |
|  | 23d | Discuss implications of the results for practice, policy, and future research. | Manuscript: discussion |
| **OTHER INFORMATION** | | | |
| Registration and protocol | 24a | Provide registration information for the review, including register name and registration number, or state that the review was not registered. | Manuscript: methods |
|  | 24b | Indicate where the review protocol can be accessed, or state that a protocol was not prepared. | Manuscript: methods |
|  | 24c | Describe and explain any amendments to information provided at registration or in the protocol. | N/A |
| Support | 25 | Describe sources of financial or non-financial support for the review, and the role of the funders or sponsors in the review. | Manuscript: methods |
| Competing interests | 26 | Declare any competing interests of review authors. | Manuscript: declaration of interests |
| Availability of data, code and other materials | 27 | Report which of the following are publicly available and where they can be found: template data collection forms; data extracted from included studies; data used for all analyses; analytic code; any other materials used in the review. | Manuscript: methods, data sharing |

# **B. Enhancing Transparency in Reporting the Synthesis of Qualitative Research (ENTREQ) checklist**

| **Item** | **Guide and description** | **Report location** |
| --- | --- | --- |
| 1. Aim | State the research question the synthesis addresses | Manuscript: introduction |
| 2. Synthesis methodology | Identify the synthesis methodology or theoretical framework which underpins the synthesis, and describe the rationale for choice of methodology (e.g. meta- ethnography, thematic synthesis, critical interpretive synthesis, grounded theory synthesis, realist synthesis, meta-aggregation, meta-study, framework synthesis) | Manuscript: methods |
| 3. Approach to searching | Indicate whether the search was pre-planned (comprehensive search strategies to seek all available studies) or iterative (to seek all available concepts until they theoretical saturation is achieved) | Manuscript: methods  Appendix C |
| 4. Inclusion criteria | Specify the inclusion/exclusion criteria (e.g. in terms of population, language, year limits, type of publication, study type) | Manuscript: methods, table 1 |
| 5. Data sources | Describe the information sources used (e.g. electronic databases (MEDLINE, EMBASE, CINAHL, psycINFO), grey literature databases (digital thesis, policy reports), relevant organisational websites, experts, information specialists, generic web searches (Google Scholar) hand searching, reference lists) and when the searches conducted; provide the rationale for using the data sources | Manuscript: methods |
| 6. Electronic Search strategy | Describe the literature search (e.g. provide electronic search strategies with population terms, clinical or health topic terms, experiential or social phenomena related terms, filters for qualitative research, and search limits) | Appendix C |
| 7. Study screening methods | Describe the process of study screening and sifting (e.g. title, abstract and full text review, number of independent reviewers who screened studies) | Manuscript: methods |
| 8. Study characteristics | Present the characteristics of the included studies (e.g. year of publication, country, population, number of participants, data collection, methodology, analysis, research questions) | Manuscript: results, table 3 |
| 9. Study selection results | Identify the number of studies screened and provide reasons for study exclusion (e.g. for comprehensive searching, provide numbers of studies screened and reasons for exclusion indicated in a figure/flowchart; for iterative searching describe reasons for study exclusion and inclusion based on modifications to the research question and/or contribution to theory development) | Manuscript: results, figure 1  Appendix D |
| 10. Rationale for appraisal | Describe the rationale and approach used to appraise the included studies or selected findings (e.g. assessment of conduct (validity and robustness), assessment of reporting (transparency), assessment of content and utility of the findings) | Manuscript: methods |
| 11. Appraisal items | State the tools, frameworks and criteria used to appraise the studies or selected findings (e.g. Existing tools: CASP, QARI, COREQ, Mays and Pope [25]; reviewer developed tools; describe the domains assessed: research team, study design, data analysis and interpretations, reporting) | Manuscript: methods |
| 12.Appraisal process | Indicate whether the appraisal was conducted independently by more than one reviewer and if consensus was required | Manuscript: methods |
| 13.Appraisal results | Present results of the quality assessment and indicate which articles, if any, were weighted/excluded based on the assessment and give the rationale | Manuscript: results  Appendix E |
| 14. Data extraction | Indicate which sections of the primary studies were analyzed and how were the data extracted from the primary studies? (e.g. all text under the headings “results /conclusions” were extracted electronically and entered into a computer software) | Manuscript: methods |
| 15. Software | State the computer software used, if any | Manuscript: methods |
| 16. Number of reviewers | Identify who was involved in coding and analysis | Manuscript: methods, table 2 |
| 17. Coding | Describe the process for coding of data (e.g. line by line coding to search for concepts) | Manuscript: methods, table 2 |
| 18. Study comparison | Describe how were comparisons made within and across studies (e.g. subsequent studies were coded into pre-existing concepts, and new concepts were created when deemed necessary) | Manuscript: methods, table 2 |
| 19. Derivation of themes | Explain whether the process of deriving the themes or constructs was inductive or deductive | Manuscript: methods, table 2 |
| 20. Quotations | Provide quotations from the primary studies to illustrate themes/constructs, and identify whether the quotations were participant quotations of the author’s interpretation | Manuscript: results |
| 21. Synthesis output | Present rich, compelling and useful results that go beyond a summary of the primary studies (e.g. new interpretation, models of evidence, conceptual models, analytical framework, development of a new theory or construct) | Manuscript: results |

# **C. Search strategy for selected bibliographic databases**

**Panel C1. Search terms for Ovid MEDLINE**

| 1 | wom?n.tw. | 1463808 |
| --- | --- | --- |
| 2 | female$.tw. | 1287428 |
| 3 | exp Women/ or exp Women, Working/ | 46477 |
| 4 | informal work*.tw. | 433 |
| 5 | informal employ*.tw. | 155 |
| 6 | informal economy.tw. | 183 |
| 7 | informal sector.tw. | 742 |
| 8 | precari* work*.tw. | 299 |
| 9 | informal enterprise.tw. | 3 |
| 10 | informal vend*.tw. | 24 |
| 11 | street vend*.tw. | 432 |
| 12 | street trad*.tw. | 26 |
| 13 | hawker.tw. | 115 |
| 14 | waste work*.tw. | 286 |
| 15 | waste pick*.tw. | 161 |
| 16 | domestic work*.tw. | 740 |
| 17 | entrepreneur.tw. | 644 |
| 18 | sex work*.tw. | 8101 |
| 19 | gig work*.tw. | 61 |
| 20 | gig economy.tw. | 85 |
| 21 | platform work*.tw. | 115 |
| 22 | platform economy.tw. | 38 |
| 23 | exp Informal Sector/ or exp Developing Countries/ or exp Employment/ | 184479 |
| 24 | covid-19.tw. | 376088 |
| 25 | covid19.tw. | 2741 |
| 26 | covid.tw. | 383268 |
| 27 | pandemic.tw. | 241414 |
| 28 | lockdown.tw. | 19217 |
| 29 | coronavirus.tw. | 137159 |
| 30 | corona virus.tw. | 3470 |
| 31 | exp COVID-19/ | 279091 |
| 32 | health*.tw. | 3935725 |
| 33 | wellbeing.tw. | 34236 |
| 34 | well-being.tw. | 131678 |
| 35 | experienc*.tw. | 1555692 |
| 36 | perspective.tw. | 327320 |
| 37 | opinion.tw. | 98969 |
| 38 | view.tw. | 360982 |
| 39 | insight.tw. | 300597 |
| 40 | impact*.tw. | 1828324 |
| 41 | outcome.tw. | 1341399 |
| 42 | 1 or 2 or 3 | 2605223 |
| 43 | 4 or 5 or 6 or 7 or 8 or 9 or 10 or 11 or 12 or 13 or 14 or 15 or 16 or 17 or 18 or 19 or 20 or 21 or 22 or 23 | 195496 |
| 44 | 24 or 25 or 26 or 27 or 28 or 29 or 30 or 31 | 480382 |
| 45 | 32 or 33 or 34 or 35 or 36 or 37 or 38 or 39 or 40 or 41 | 7904538 |
| 46 | 42 and 43 and 44 and 45 | 643 |
| 47 | limit 46 to yr="2020 -Current" | 567 |
| 48 | limit 47 to english language | 552 |

**Panel C2. Search syntax adapted to other databases**

| **Database** | **Search terms** | **Filter** | **Results** |
| --- | --- | --- | --- |
| Web of Science | (ALL=Women OR ALL="women, working" OR (TI=Women OR AB=Women) OR (TI=female OR AB=female))  AND  (ALL="Informal Sector" OR ALL="Developing Countries" OR ALL=Employment OR (TI="informal work*" OR AB="informal work*") OR (TI="informal employ*" OR AB="informal employ*") OR (TI="informal economy" OR AB="informal economy") OR (TI="Informal Sector" OR AB="Informal Sector") OR (TI="precar* work*" OR AB="precar* work*") OR (TI="informal enterprise" OR AB="informal enterprise") OR (TI="informal vend*" OR AB="informal vend*") OR (TI="street vend*" OR AB="street vend*") OR (TI="street trad*" OR AB="street trad*") OR (TI=hawker OR AB=hawker) OR (TI="domestic work*" OR AB="domestic work*") OR (TI="waste work*" OR AB="waste work*") OR (TI="waste pick*" OR AB="waste pick*") OR (TI=entrepreneur OR AB=entrepreneur) OR (TI="sex work*" OR AB="sex work*") OR (TI="gig work*" OR AB="gig work*") OR (TI="gig economy" OR AB="gig economy") OR (TI="platform work*" OR AB="platform work*") OR (TI="platform economy" OR AB="platform economy"))  AND  (ALL="covid 19" OR (TI="covid 19" OR AB="covid 19") OR (TI=covid19 OR AB=covid19) OR (TI=covid OR AB=covid) OR (TI=pandemic OR AB=pandemic) OR (TI=lockdown OR AB=lockdown) OR (TI=coronavirus OR AB=coronavirus) OR (TI="corona virus" OR AB="corona virus"))  AND  ((TI=health* OR AB=health*) OR (TI=wellbeing OR AB=wellbeing) OR (TI=well-being OR AB=well-being) OR (TI=experienc* OR AB=experienc*) OR (TI=perspective OR AB=perspective) OR (TI=opinion OR AB=opinion) OR (TI=view OR AB=view) OR (TI=insight OR AB=insight) OR (TI=impact* OR AB=impact*) OR (TI=outcome OR AB=outcome)) | Publication date 2020-current;  English language | 1870 |
| Scopus | ( INDEXTERMS ( women ) OR INDEXTERMS ( "women, working" ) OR ( TITLE-ABS ( women ) OR TITLE-ABS ( female ) ) )  AND  ( INDEXTERMS ( "Informal Sector" ) OR INDEXTERMS ( "Developing Countries" ) OR INDEXTERMS ( employment ) OR ( TITLE-ABS ( "informal work*" ) OR TITLE-ABS ( "informal employ*" ) OR TITLE-ABS ( "informal economy" ) OR TITLE-ABS ( "Informal Sector" ) OR TITLE-ABS ( "precar* work*" ) OR TITLE-ABS ( "informal enterprise" ) OR TITLE-ABS ( "informal vend*" ) OR TITLE-ABS ( "street vend*" ) OR TITLE-ABS ( "street trad*" ) OR TITLE-ABS ( hawker ) OR TITLE-ABS ( "domestic work*" ) OR TITLE-ABS ( "waste work*" ) OR TITLE-ABS ( "waste pick*" ) OR TITLE-ABS ( entrepreneur ) OR TITLE-ABS ( "sex work*" ) OR TITLE-ABS ( "gig work*" ) OR TITLE-ABS ( "gig economy" ) OR TITLE-ABS ( "platform work*" ) OR TITLE-ABS ( "platform economy" ) ) )  AND  ( INDEXTERMS ( "covid 19" ) OR ( TITLE-ABS ( "covid 19" ) OR TITLE-ABS ( covid19 ) OR TITLE-ABS ( covid ) OR TITLE-ABS ( pandemic ) OR TITLE-ABS ( lockdown ) OR TITLE-ABS ( coronavirus ) OR TITLE-ABS ( "corona virus" ) ) )  AND  ( TITLE-ABS ( health* ) OR TITLE-ABS ( wellbeing ) OR TITLE-ABS ( well-being ) OR TITLE-ABS ( experienc* ) OR TITLE-ABS ( perspective ) OR TITLE-ABS ( opinion ) OR TITLE-ABS ( view ) OR TITLE-ABS ( insight ) OR TITLE-ABS ( impact* ) OR TITLE-ABS ( outcome ) ) | Publication date 2020-current;  English language | 1730 |
| PsycINFO | (exp "Human Females"/ or exp "Working Women"/ or (women or female).ti,ab.)  and  (exp Occupations/ or exp "Employment Status"/ or exp "Developing Countries"/ or ("informal work*" or "informal employ*" or "informal economy" or "Informal Sector" or "precar* work*" or "informal enterprise" or "informal vend*" or "street vend*" or "street trad*" or hawker or "domestic work*" or "waste work*" or "waste pick*" or entrepreneur or "sex work*" or "gig work*" or "gig economy" or "platform work*" or "platform economy").ti,ab.)  and  (exp COVID-19/ or ("covid 19" or covid19 or covid or pandemic or lockdown or coronavirus or "corona virus").ti,ab.)  and  (health or wellbeing or well-being or experienc* or perspective or opinion or view or insight or impact* or outcome).ti,ab. | Publication date 2020-current;  English language | 247 |

# **D. Excluded studies**

| **Item #** | **Study ID**  **(First author, Publication year)** | | **Title** | **Reason for exclusion** |
| --- | --- | --- | --- | --- |
| 1 | Abdukadyrova | 2023 | “The pandemic played a cruel joke on us”: the vulnerabilities of Kyrgyz women migrant workers in Russia during COVID-19 | Unable to distinguish data from women informal worker |
| 2 | Abuhussein | 2023 | The impact of COVID-19 on refugee women's entrepreneurship in Jordan | Unable to distinguish data from women informal worker |
| 3 | Akbas | 2021 | Women's health anxiety and psychological wellbeing during the COVID-19 pandemic. A descriptive study | Data not collected using interviews, focus groups, or observation |
| 4 | Anderson | 2021 | Staying home, distancing, and face masks: COVID-19 prevention among U.S. women in the cope study | Data not collected using interviews, focus groups, or observation |
| 5 | Anjali Anwar | 2022 | Gig platforms as faux infrastructure: a case study of women beauty workers in India | Unrelated to health and wellbeing experiences |
| 6 | Banta | 2023 | Immobilised by the pandemic: Filipino domestic workers and seafarers in the time of COVID-19 | Unable to distinguish data from women informal worker |
| 7 | Banu | 2023 | Impact of COVID-19 pandemic on livelihoods of informal workers in Kolkata: from sustainable livelihood perspective | Unable to distinguish data from women informal worker |
| 8 | Barik | 2024 | The impact of COVID-19 on women micro-entrepreneurs in Odisha: a case study of Cuttack | Unable to distinguish data from women informal worker |
| 9 | Bhattacharjee | 2023 | A gendered approach to examining pandemic-induced livelihood crisis in the informal sector: the case of female domestic workers in Titwala | Unrelated to health and wellbeing experiences |
| 10 | Binu Sahayam | 2022 | Desperation and panic grief of women migrants during Covid 19 pandemic | Unable to distinguish data from women informal worker |
| 11 | Bofill-Poch | 2021 | “You have nowhere to go (and I do)”. How fear of contagion affects migrant domestic workers | Full text report is not available in English |
| 12 | Boo | 2021 | Unpaid domestic work and gender inequality in the time of COVID-19 in Malaysia | Data not collected using interviews, focus groups, or observation |
| 13 | Brown | 2022 | Changes in life circumstances and mental health symptoms during the COVID-19 pandemic among midlife women with elevated risk for cardiovascular disease | Data not collected using interviews, focus groups, or observation |
| 14 | Cámbara | 2022 | COVID-19 and women migrant workers in informal employment: recommendations for strengthening social protection efforts in Lao People’s Democratic Republic | Data not collected using interviews, focus groups, or observation |
| 15 | Castillón | 2021 | Pandemic and care: responses from the self-organization of domestic workers | Full text report is not available in English |
| 16 | Chela-Alvarez | 2022 | Experiences and concerns of female hotel housekeepers in the first stages of the Covid-19 lockdown in the Balearic Islands (Spain): a qualitative study | Unable to distinguish data from women informal worker |
| 17 | Chen | 2022 | COVID-19 and informal work: evidence from 11 cities | Unable to distinguish data from women informal worker |
| 18 | Couto | 2023 | Self-care from the perspective by female sex workers to prevent and face the SARS-CoV-2 pandemic | Repeat publications from the same study |
| 19 | de Diego-Cordero | 2022 | COVID-19 and female immigrant caregivers in Spain: cohabiting during lockdown | Unable to distinguish data from women informal worker |
| 20 | Dempere | 2023 | The impact of COVID-19 on women's empowerment: a global perspective | Data not collected using interviews, focus groups, or observation |
| 21 | Ebuenyi | 2022 | Unemployment in women with psychosocial disabilities during the COVID-19 pandemic: lessons from Tana River County, Kenya | Unable to distinguish data from women informal worker |
| 22 | Fleischer | 2023 | Home, shelter, trap experiences of pandemic confinement in Bogotá, Colombia | Unable to distinguish data from women informal worker |
| 23 | Francisco-Menchavez | 2023 | Filipina caregivers and mental health under COVID-19: Impacts of transnational obligations and precarious work on migrant care workers in the United States of America | Unable to distinguish data from women informal worker |
| 24 | Granger | 2022 | Adapting to a jolt: a mixed methods study identifying challenges and personal resources impacting professional gig workers' well-being during COVID-19 | Data not collected using interviews, focus groups, or observation |
| 25 | Harrison | 2022 | Support from friends moderates the relationship between repetitive negative thinking and postnatal wellbeing during COVID-19 | Unable to distinguish data from women informal worker |
| 26 | Hartmann | 2022 | The forgotten essential workers in the circular economy? Waste picker precarity and resilience amidst the COVID-19 pandemic | Unable to distinguish data from women informal worker |
| 27 | Hassan | 2023 | Informal settlements, Covid-19 and sex workers in Kenya | Repeat publications from the same study |
| 28 | Hungwe | 2024 | Effects of COVID-19 on the livelihoods of women with disabilities in Zimbabwe: a study of three low-income areas in Harare Metropolitan Province | Unable to distinguish data from women informal worker |
| 29 | Iglesias-Rios | 2024 | Precarious work and housing for Michigan farmworkers during the COVID-19 pandemic and beyond | Unrelated to the pandemic context |
| 30 | Intesar | 2024 | Living with vulnerability: triple burden through the eyes of urban slum women in Bangladesh | Unrelated to the pandemic context |
| 31 | Ismail | 2022 | COVID-19 impact and recovery for women informal workers - a view from 2021 | Unrelated to health and wellbeing experiences |
| 32 | Jahanshahi | 2023 | Unveiling resilience: strategic responses, success factors, and challenges faced by Latin American female entrepreneurs amid the COVID-19 pandemic | Data not collected using interviews, focus groups, or observation |
| 33 | Jangir | 2022 | Impact of pandemic on women engaged in bar dances and sex work: a case of Nat community in Rajasthan | Unable to distinguish data from women informal worker |
| 34 | Josyula | 2022 | Isolation in COVID, and COVID in isolation - exacerbated shortfalls in provision for women's health and well-being among marginalized urban communities in India | Data not collected using interviews, focus groups, or observation |
| 35 | Khursheed | 2021 | Turning the COVID-19 crisis into entrepreneurial success: an exploratory study on women innovators of Pakistan | Unable to distinguish data from women informal worker |
| 36 | Lafferty | 2022 | Colliding worlds: family carers' experiences of balancing work and care in Ireland during the COVID-19 pandemic | Unable to distinguish data from women informal worker |
| 37 | Markose | 2023 | COVID-19 and women in the tourism & hospitality workforce: a thematic analysis | Unable to distinguish data from women informal worker |
| 38 | Martins | 2023 | Negotiating multiple risks: health, safety, and well-being among internal migrant sex workers in Brazil during COVID-19 | Unable to distinguish data from women informal worker |
| 39 | Mathew | 2020 | The impact of COVID-19 lockdown in a developing country: narratives of self-employed women in Ndola, Zambia | Unable to distinguish data from women informal worker |
| 40 | Mira | 2023 | Mexican women narratives facing Covid-19: job insecurity and family overload | Full text report is not available in English |
| 41 | Mlambo | 2022 | We are human beings: the social support to commercial sex workers during COVID-19 in the criminalised setting at uMhlathuze Local Municipality, KwaZulu-Natal, South Africa | Repeat publications from the same study |
| 42 | Modisaotsile | 2023 | Heightened risk of unintended pregnancy among sex workers and sex worker organizations' response during the stringent COVID-19 containment measures in East and Southern Africa | Unable to distinguish data from women informal worker |
| 43 | More | 2022 | The growth of precarious employment for women in the care work sector during the COVID-19 pandemic | Unable to distinguish data from women informal worker |
| 44 | Moyo | 2022 | Utilisation of HIV services by female sex workers in Zimbabwe during the COVID-19 pandemic: a descriptive phenomenological study | Repeat publications from the same study |
| 45 | Muhammad | 2023 | The impact of the COVID-19 pandemic on women entrepreneurs in Pakistan | Data not collected using interviews, focus groups, or observation |
| 46 | Muzuva | 2022 | Impact of COVID 19 on livelihoods of female entrepreneurs in Marondera urban | Unable to distinguish data from women informal worker |
| 47 | Poirier | 2022 | Informality, social citizenship, and wellbeing among migrant workers in Costa Rica in the context of COVID-19 | Unable to distinguish data from women informal worker |
| 48 | Premji | 2024 | The health and safety experiences of precariously employed Bangladeshi immigrant workers in Toronto during the COVID-19 pandemic | Unable to distinguish data from women informal worker |
| 49 | Rahman | 2023 | COVID-19 induced impacts on women workers of fish and shellfish processing plants in Bangladesh | Unable to distinguish data from women informal worker |
| 50 | Rahmani | 2024 | Challenges of the COVID-19 pandemic on women’s HIV harm reduction centers: a mixed-methods study | Unable to distinguish data from women informal worker |
| 51 | Rajan | 2024 | Impact of COVID-19 on women migrant workers: case of domestic workers in the South Asia-Gulf corridor | Unrelated to health and wellbeing experiences |
| 52 | Rico | 2021 | Migrant domestic work in Chile and COVID-19. Bolivian caregivers on waste ground | Full text report is not available in English |
| 53 | Saxena | 2023 | Gender and disruptions in family routines and stress amid COVID-19 | Data not collected using interviews, focus groups, or observation |
| 54 | Shahrullah | 2024 | Evaluating women's economic empowerment during COVID-19 pandemic in South Sulawesi, Indonesia | Unable to distinguish data from women informal worker |
| 55 | Teixeira | 2024 | Responses of workers' organizations to the COVID-19 crisis: intersectional approaches of domestic workers in Mexico | Unable to distinguish data from women informal worker |
| 56 | Thanh | 2022 | The COVID-19 pandemic and the livelihood of a vulnerable population: evidence from women street vendors in urban Vietnam | Unrelated to health and wellbeing experiences |
| 57 | The Duy | 2024 | Economic challenges faced by migrant street vendors during a crisis: implications for social inclusion development | Unable to distinguish data from women informal worker |
| 58 | Vega | 2021 | “Echarnos la mano”. Organizational experiences pertaining to life sustainability of migrant Mazatec women working in domestic settings in popular neighborhoods of Puebla, Mexico | Full text report is not available in English |
| 59 | Wichit | 2023 | Shan female sex workers' vulnerabilities and coping strategies in response to COVID-19 in Chiang Mai, Thailand | Unable to distinguish data from women informal worker |
| 60 | Winata | 2023 | Therapeutic landscapes, networks, and health and wellbeing during the COVID-19 pandemic: a mixed-methods study among female domestic workers | Unable to distinguish data from women informal worker |
| 61 | Xue | 2021 | Gender differences in unpaid care work and psychological distress in the UK Covid-19 lockdown | Data not collected using interviews, focus groups, or observation |
| 62 | Zhanda | 2022 | Women in the informal sector amid COVID-19: Implications for household peace and economic stability in urban Zimbabwe | Data not collected using interviews, focus groups, or observation |
| 63 | Zuma | 2021 | Lives interrupted: navigating hardship during COVID-19 provides lessons in solidarity and visibility for mobile young people in South Africa and Uganda | Repeat publications from the same study |

# **E. Overview of quality assessment using Mixed Methods Appraisal Tool**

|  | **S1** | **S2** |  | **1.1** | **1.2** | **1.3** | **1.4** | **1.5** |  | **2.1** | **2.2** | **2.3** | **2.4** | **2.5** |  | **3.1** | **3.2** | **3.3** | **3.4** | **3.5** |
| --- | --- | --- | --- | --- | --- | --- | --- | --- | --- | --- | --- | --- | --- | --- | --- | --- | --- | --- | --- | --- |
| **Articles reporting qualitative research**  **First author (Publication year)** | | | | | | | | | | | | | | | | | | | | |
| Aantjes  (2022) |  |  |  |  |  |  |  |  |  |  |  |  |  |  |  |  |  |  |  |  |
| Agha  (2022) |  |  |  |  |  |  |  |  |  |  |  |  |  |  |  |  |  |  |  |  |
| AleAhmad  (2023) |  |  |  |  |  |  |  |  |  |  |  |  |  |  |  |  |  |  |  |  |
| Arora  (2021) |  |  |  |  |  |  |  |  |  |  |  |  |  |  |  |  |  |  |  |  |
| Asriani  (2021) |  |  |  |  |  |  |  |  |  |  |  |  |  |  |  |  |  |  |  |  |
| Azeez E P  (2021) |  |  |  |  |  |  |  |  |  |  |  |  |  |  |  |  |  |  |  |  |
| Babu  (2024) |  |  |  |  |  |  |  |  |  |  |  |  |  |  |  |  |  |  |  |  |
| Banerjee  (2024) |  |  |  |  |  |  |  |  |  |  |  |  |  |  |  |  |  |  |  |  |
| Barhoi  (2024) |  |  |  |  |  |  |  |  |  |  |  |  |  |  |  |  |  |  |  |  |
| Belete  (2020) [preprint] |  |  |  |  |  |  |  |  |  |  |  |  |  |  |  |  |  |  |  |  |
| Bishop  (2024) |  |  |  |  |  |  |  |  |  |  |  |  |  |  |  |  |  |  |  |  |
| Boateng-Pobee  (2021) |  |  |  |  |  |  |  |  |  |  |  |  |  |  |  |  |  |  |  |  |
| Bossenbroek  (2021) |  |  |  |  |  |  |  |  |  |  |  |  |  |  |  |  |  |  |  |  |
| Burgos  (2021) |  |  |  |  |  |  |  |  |  |  |  |  |  |  |  |  |  |  |  |  |
| Cabras  (2022) |  |  |  |  |  |  |  |  |  |  |  |  |  |  |  |  |  |  |  |  |
| Cadogan-McClean  (2023) |  |  |  |  |  |  |  |  |  |  |  |  |  |  |  |  |  |  |  |  |
| Couto  (2022) |  |  |  |  |  |  |  |  |  |  |  |  |  |  |  |  |  |  |  |  |
| Crankshaw  (2023) |  |  |  |  |  |  |  |  |  |  |  |  |  |  |  |  |  |  |  |  |
| de Silva  (2022) |  |  |  |  |  |  |  |  |  |  |  |  |  |  |  |  |  |  |  |  |
| Dogar  (2022) |  |  |  |  |  |  |  |  |  |  |  |  |  |  |  |  |  |  |  |  |
| Fabbri  (2022) |  |  |  |  |  |  |  |  |  |  |  |  |  |  |  |  |  |  |  |  |
| Gichuna  (2020) |  |  |  |  |  |  |  |  |  |  |  |  |  |  |  |  |  |  |  |  |
| Hattar  (2023) |  |  |  |  |  |  |  |  |  |  |  |  |  |  |  |  |  |  |  |  |
| King  (2023) |  |  |  |  |  |  |  |  |  |  |  |  |  |  |  |  |  |  |  |  |
| Mapuranga  (2021) |  |  |  |  |  |  |  |  |  |  |  |  |  |  |  |  |  |  |  |  |
| Mavhandu-Mudzusi  (2022) |  |  |  |  |  |  |  |  |  |  |  |  |  |  |  |  |  |  |  |  |
| Mbombo  (2022) |  |  |  |  |  |  |  |  |  |  |  |  |  |  |  |  |  |  |  |  |
| Mlambo  (2023) |  |  |  |  |  |  |  |  |  |  |  |  |  |  |  |  |  |  |  |  |
| Mohmand  (2023) |  |  |  |  |  |  |  |  |  |  |  |  |  |  |  |  |  |  |  |  |
| Mustafa  (2021) |  |  |  |  |  |  |  |  |  |  |  |  |  |  |  |  |  |  |  |  |
| Muswede  (2022) |  |  |  |  |  |  |  |  |  |  |  |  |  |  |  |  |  |  |  |  |
| Nhiwatiwa  (2023) |  |  |  |  |  |  |  |  |  |  |  |  |  |  |  |  |  |  |  |  |
| Nyabeze  (2022) |  |  |  |  |  |  |  |  |  |  |  |  |  |  |  |  |  |  |  |  |
| Oyebamiji  (2023) |  |  |  |  |  |  |  |  |  |  |  |  |  |  |  |  |  |  |  |  |
| Parlak  (2022) |  |  |  |  |  |  |  |  |  |  |  |  |  |  |  |  |  |  |  |  |
| Singh  (2022) |  |  |  |  |  |  |  |  |  |  |  |  |  |  |  |  |  |  |  |  |
| Sithole  (2022) |  |  |  |  |  |  |  |  |  |  |  |  |  |  |  |  |  |  |  |  |
| Tshivhase  (2023) |  |  |  |  |  |  |  |  |  |  |  |  |  |  |  |  |  |  |  |  |
| Wasima  (2022) |  |  |  |  |  |  |  |  |  |  |  |  |  |  |  |  |  |  |  |  |
| Yadav  (2024) |  |  |  |  |  |  |  |  |  |  |  |  |  |  |  |  |  |  |  |  |
| Zulfiqar  (2022) |  |  |  |  |  |  |  |  |  |  |  |  |  |  |  |  |  |  |  |  |
| **Articles reporting mixed methods research***  **First author (Publication year)** | | | | | | | | | | | | | | | | | | | | |
| Abrefa Busia  (2023) |  |  |  |  |  |  |  |  |  |  |  |  |  |  |  |  |  |  |  |  |
| Ajuwon  (2021) |  |  |  |  |  |  |  |  |  |  |  |  |  |  |  |  |  |  |  |  |
| Balampama  (2023) |  |  |  |  |  |  |  |  |  |  |  |  |  |  |  |  |  |  |  |  |
| Bhat  (2021) |  |  |  |  |  |  |  |  |  |  |  |  |  |  |  |  |  |  |  |  |
| Callander  (2022) |  |  |  |  |  |  |  |  |  |  |  |  |  |  |  |  |  |  |  |  |
| Ogando  (2022) |  |  |  |  |  |  |  |  |  |  |  |  |  |  |  |  |  |  |  |  |
| Samudyatha  (2024) |  |  |  |  |  |  |  |  |  |  |  |  |  |  |  |  |  |  |  |  |
| Sumalatha  (2021) |  |  |  |  |  |  |  |  |  |  |  |  |  |  |  |  |  |  |  |  |
| Vyas  (2023) |  |  |  |  |  |  |  |  |  |  |  |  |  |  |  |  |  |  |  |  |
| Wandera  (2023a) |  |  |  |  |  |  |  |  |  |  |  |  |  |  |  |  |  |  |  |  |
| Wandera  (2023b) |  |  |  |  |  |  |  |  |  |  |  |  |  |  |  |  |  |  |  |  |
| Wrigley-Asante  (2024) |  |  |  |  |  |  |  |  |  |  |  |  |  |  |  |  |  |  |  |  |

|  |  | Yes |  |  | Can’t tell |  |  | No |
| --- | --- | --- | --- | --- | --- | --- | --- | --- |

## Panel E1 Methodological quality criteria questions corresponding to study design based on Mixed Methods Appraisal Tool checklist

| Study design | Methodological quality criteria | |
| --- | --- | --- |
| All types | S1 | Are there clear research questions? |
|  | S2 | Do the collected data allow to address the research questions? |
| 1. Qualitative | 1.1 | Is the qualitative approach appropriate to answer the research question? |
|  | 1.2 | Are the qualitative data collection methods adequate to address the research question? |
|  | 1.3 | Are the findings adequately derived from the data? |
|  | 1.4 | Is the interpretation of results sufficiently substantiated by data? |
|  | 1.5 | Is there coherence between qualitative data sources, collection, analysis and interpretation? |
| 2. Quantitative descriptive | 2.1 | Is the sampling strategy relevant to address the research question? |
|  | 2.2 | Is the sample representative of the target population? |
|  | 2.3 | Are the measurements appropriate? |
|  | 2.4 | Is the risk of nonresponse bias low? |
|  | 2.5 | Is the statistical analysis appropriate to answer the research question? |
| 3. Mixed methods | 3.1 | Is there an adequate rationale for using a mixed methods design to address the research question? |
|  | 3.2 | Are the different components of the study effectively integrated to answer the research question? |
|  | 3.3 | Are the outputs of the integration of qualitative and quantitative components adequately interpreted? |
|  | 3.4 | Are divergences and inconsistencies between quantitative and qualitative results adequately addressed? |
|  | 3.5 | Do the different components of the study adhere to the quality criteria of each tradition of the methods involved? |
| **Quantitative components of all mixed methods research included in this review were descriptive.* | | |

# **F. Overview of themes contained in included studies**

|  | **Theme 1:**  **Exacerbation of existing vulnerabilities** | | | |  | **Theme 2:**  **Negotiation of risks and resilience** | | | |  | **Theme 3:**  **Interconnectedness of health and wellbeing stressors** | | | | | |  | **Theme 4:**  **Variable experiences across social locations** | | | |
| --- | --- | --- | --- | --- | --- | --- | --- | --- | --- | --- | --- | --- | --- | --- | --- | --- | --- | --- | --- | --- | --- |
| **First author**  **(Publication year)** | Precarious work | Household poverty | Patriarchal society | Stigma and discrimination |  | Mixed patterns of belief and protective behaviours | Risk and effect of infection | Exposure to harm | Coping strategies |  | Financial strain | Physical and mental health | Shelter and food security | Interpersonal tension | Barriers to healthcare | Health behaviour |  | Sex workers | Migrant women | Identity categories | Positive experiences |
| Aantjes  (2022) | X | X | X | X |  | X |  | X | X |  | X | X | X | X |  |  |  | X | X | X |  |
| Abrefa Busia  (2023) | X |  |  |  |  |  |  |  | X |  | X | X |  |  |  |  |  |  |  |  | X |
| Agha  (2022) | X | X | X | X |  | X |  | X | X |  | X |  | X |  |  |  |  |  |  |  |  |
| Ajuwon  (2021) | X |  |  |  |  | X |  |  | X |  | X |  |  |  |  |  |  |  |  |  |  |
| AleAhmad  (2023) | X | X | X |  |  | X |  | X | X |  | X | X | X | X | X |  |  |  |  | X |  |
| Arora  (2021) | X |  | X | X |  | X | X | X | X |  | X | X | X | X |  |  |  |  | X | X |  |
| Asriani  (2021) | X | X | X |  |  | X |  |  | X |  | X | X |  | X |  |  |  |  |  |  |  |
| Azeez E P  (2021) | X | X | X | X |  | X | X | X | X |  | X | X | X |  | X |  |  |  | X |  |  |
| Babu  (2024) | X |  |  | X |  | X |  | X | X |  | X | X | X | X | X | X |  | X |  | X | X |
| Balampama  (2023) | X |  |  |  |  | X | X | X | X |  | X |  | X |  |  |  |  |  |  |  |  |
| Banerjee  (2024) | X |  | X | X |  | X | X | X |  |  | X | X | X | X | X |  |  |  | X | X |  |
| Barhoi  (2024) | X |  | X | X |  |  |  | X |  |  | X |  |  | X |  |  |  |  |  | X |  |
| Belete  (2020) [preprint] | X |  |  | X |  |  | X | X | X |  | X | X | X |  |  | X |  | X |  |  |  |
| Bhat  (2021) |  |  | X | X |  | X | X | X | X |  | X | X | X | X | X |  |  |  |  |  |  |
| Bishop  (2024) | X |  |  |  |  | X |  |  | X |  | X | X |  |  |  |  |  |  |  | X | X |
| Boateng-Pobee  (2021) | X |  | X |  |  | X | X |  | X |  | X | X | X | X |  |  |  |  |  | X |  |
| Bossenbroek  (2021) | X |  | X |  |  | X | X | X | X |  | X | X | X |  | X |  |  |  |  | X |  |
| Burgos  (2021) | X |  |  |  |  | X | X | X | X |  | X | X | X | X |  |  |  |  | X | X |  |
| Cabras  (2022) | X |  | X |  |  | X | X | X | X |  | X | X |  | X |  |  |  |  | X |  |  |
| Cadogan-McClean  (2023) | X |  | X |  |  | X | X |  | X |  | X | X |  |  |  |  |  |  |  | X | X |
| Callander  (2022) | X |  |  | X |  | X |  | X | X |  | X |  | X | X | X | X |  | X | X | X |  |
| Couto  (2022) | X |  |  | X |  | X | X |  | X |  | X | X |  | X |  | X |  | X |  |  |  |
| Crankshaw  (2023) | X |  |  | X |  |  |  | X | X |  | X |  | X |  |  |  |  | X |  | X |  |
| de Silva  (2022) | X |  | X |  |  | X | X |  | X |  | X |  |  |  |  |  |  |  |  | X | X |
| Dogar  (2022) | X | X |  |  |  |  |  | X | X |  | X | X | X | X | X |  |  |  |  | X |  |
| Fabbri  (2022) | X |  |  |  |  | X | X | X | X |  | X | X | X |  | X | X |  | X | X | X |  |
| Gichuna  (2020) | X |  |  | X |  | X |  | X | X |  | X |  | X |  | X | X |  | X |  | X |  |
| Hattar  (2023) | X |  | X | X |  |  |  | X | X |  | X |  | X | X |  |  |  |  | X | X | X |
| King  (2023) | X | X | X |  |  | X |  | X | X |  | X | X | X | X | X | X |  |  |  | X |  |
| Mapuranga  (2021) | X |  |  |  |  | X |  |  | X |  | X | X |  | X |  |  |  |  | X |  |  |
| Mavhandu-Mudzusi  (2022) | X |  |  | X |  | X |  | X | X |  | X | X | X |  |  | X |  | X |  |  |  |
| Mbombo  (2022) | X | X | X |  |  | X | X | X |  |  | X | X | X |  |  |  |  |  |  | X |  |
| Mlambo  (2023) | X |  |  | X |  | X |  | X | X |  | X |  |  |  |  |  |  | X |  |  |  |
| Mohmand  (2023) |  |  |  |  |  | X |  |  |  |  | X |  |  |  |  |  |  |  |  | X |  |
| Mustafa  (2021) | X | X | X |  |  |  |  |  | X |  | X | X | X |  | X |  |  |  |  | X |  |
| Muswede  (2022) | X |  |  |  |  | X |  |  | X |  | X | X | X |  | X |  |  |  | X |  |  |
| Nhiwatiwa  (2023) | X |  | X |  |  | X |  | X | X |  | X | X | X |  |  |  |  |  |  | X | X |
| Nyabeze  (2022) | X |  |  | X |  | X |  | X | X |  | X |  | X |  | X | X |  | X |  |  |  |
| Ogando  (2022) | X |  | X |  |  |  |  | X | X |  | X | X | X | X |  |  |  |  |  | X |  |
| Oyebamiji  (2023) | X |  |  |  |  |  |  | X | X |  | X | X | X |  |  |  |  |  | X |  |  |
| Parlak  (2022) | X |  | X |  |  | X | X | X | X |  | X | X | X | X |  |  |  |  |  |  |  |
| Samudyatha  (2024) | X |  |  |  |  | X |  | X | X |  | X |  | X | X |  | X |  | X |  |  |  |
| Singh  (2022) | X | X | X | X |  |  | X | X | X |  | X | X | X |  | X |  |  |  |  | X |  |
| Sithole  (2022) | X |  | X |  |  | X | X | X | X |  | X |  |  |  |  |  |  |  | X | X |  |
| Sumalatha  (2021) | X | X | X |  |  |  | X | X | X |  | X | X | X | X | X |  |  |  |  |  |  |
| Tshivhase  (2023) |  |  |  |  |  |  |  |  | X |  |  |  |  |  |  |  |  |  |  |  | X |
| Vyas  (2023) | X | X | X | X |  |  |  | X | X |  | X | X | X | X | X | X |  |  | X | X |  |
| Wandera  (2023a) |  |  | X |  |  | X |  |  | X |  | X |  |  |  | X |  |  |  |  |  | X |
| Wandera  (2023b) | X |  | X |  |  |  |  | X | X |  | X |  | X | X | X |  |  |  |  | X | X |
| Wasima  (2022) | X | X | X | X |  | X | X | X | X |  | X | X | X | X |  |  |  |  |  |  |  |
| Wrigley-Asante  (2024) | X |  | X |  |  |  |  | X | X |  | X | X | X |  |  |  |  |  |  | X |  |
| Yadav  (2024) | X |  |  |  |  | X | X |  |  |  | X | X | X |  | X |  |  |  | X |  |  |
| Zulfiqar  (2022) | X | X | X |  |  | X |  | X | X |  | X | X | X | X | X |  |  |  |  |  | X |

**G. Relevant extracted quotations aligning with themes and subthemes**

|  | **Themes** | **Subthemes** | **Descriptions** | **Quotations from participants in contributing study** | **Contributing studies** |
| --- | --- | --- | --- | --- | --- |
| 1 | Exacerbation of existing vulnerabilities | 1. Precarious work | The pandemic magnified the precarity of informal work for women workers who were already undermined by issues such as low wages, limited rights and weak social protection. | *“I see that this year is very diﬃcult. It is diﬃcult to live like this with a sick relative screaming from pain, rolling without a thing to eat. It’s really very hard. I have faced great diﬃculties because I am out of work. I’m out of options because of this disease really.”*  *“We suffered a lot during the lockdown. My ready-made items could not reach the market. Being a woman, I have always struggled to sell my products, but it became even harder during the lockdown. We tried hard to sell a few items so to earn some money, but all in vain.”*  *“Market is not selling and moving as before, we put things down, we didn’t see people to come and buy there is no money to spend, there are wares to sell, but people are not buying, our customers that we are calling said they are under lockdown; they are not going out too and as such no money.”*  *“I am concerned because the nature of our work involves close physical contact between two people, and doing so tends to increase the risk of transmission of this pandemic.”*  *“But you think they (employers) will keep my job till I return? Why? It’s not like no one apart from Shaalu knows how to cook or wash dishes (laughs). In the news they tell us so many people have lost their jobs, anyone of them can get my job isn’t it? With no job here (in the plantations) and there (in Delhi), what is to become of us? No news will tell you that.”*  *“We are faltu; we only get to work for a few months. But this time, our two months got wasted. In March and April, we did not get any work.”*  *“I have been working as a commercial sex worker for more than 10 years. Over this period, I have never suffered severe economic hardship like the case I am facing after the corona outbreak. It is almost two months since I met a client and earned money.”*  *“Yes, work has decreased a little because there are people who do not go out. They are afraid of the contagion and right now the contagion has increased. I work but very little, and with the curfew, it has changed too much. It has blocked our work.”*  *“COVID-19 has negatively impacted my business as I survive from hand to mouth, I no longer have the funds to buy the next stock.”*  *“Sex work was my main source of income. With the lockdown, challenges emerged as I could not access any customer. They are not available to buy our sex service. I could go for more than a week without getting any money.”*  *“I sell sweets and chips at the train station, my income was dependent on the business. However, due to the lockdown restrictions, I did not have a permit to operate during the lockdown.”*  *“I would say that in my life I have never seen such a crisis that drastically reduced our sales Undoubtedly, the purchasing power of people is reduced at a very fast rate because all businesses were severely affected due to lockdown My business was completely closed during the lockdown. I was relying on my savings.”*  *“Before the pandemic, at least customers were coming but when the pandemic came and there was restrictions and customers were affected. Customers who used to support me before the pandemic but after that all were affected; some lost their jobs, and some because of the restrictions were staying in their houses, so customers were not coming to buy.”*  *“We have financial deficiencies, but I cannot ask for it. Even under normal circumstances, we are barely getting by. I used to contribute to my spouse a little bit, but since I am not working now, we are making a living with only one salary. And this disrupted me severely. Not only me, but my children and husband as well. Because we have no money, and the prices have risen up. We lack in every way. I don’t know what will happen or for how long this process will continue.”*  *“COVID affected our businesses; even though some of us remained working, the number of customers reduced.”*  *“The lockdown began on March 20, the income accrued by homeworkers and factory workers between March 1 and March 19 was never paid. And then all work disappeared: those doing zardozi (embroidery), putting zips for local and international brands, making chappal (slippers). . .everything just stopped.”* | Aantjes (2022)  Agha (2022)  Ajuwon (2021)  Balampama (2023)  Banerjee (2022)  Barhoi (2024)  Belete (2020)  Fabbri (2022)  Mapuranga (2021)  Mavhandu-Mudzusi  (2022)  Mbombo (2022)  Mustafa (2021)  Oyebamiji (2023)  Parlak (2022)  Wandera (2023b)  Zulfiqar (2022) |
|  |  | 2. Household poverty | The pandemic intensified economic insecurity for the entire household as women informal workers often come from poor families where other members also relied on precarious employment. | *“The diﬃculty I have now is that they do not come much in the house, do not care for me. For example, yesterday I tried to call them because I did not have anything to eat, no one answered me. I was like this. We were without dinner and this afternoon my grandmother had about 40 meticais. I took that money to buy ﬂour for 20 MT, majembes, tomato and made us a roast majembes, and chima for lunch...My grandmother goes to the machamba [the ﬁeld] and I also help her. […] If there’s anything, we cook, if we don’t, we stay that way. Because of this coronavirus a lot has really changed.”*  *“We were living from hand to mouth until corona arrived. At the outset of COVID, we did not care about it because we did not know about this virus. People were living their everyday lives until we heard about the closure of the Iran–Iraq border. It was a big shock for me and others. Almost all men in our village work as Kolbars, and this is the only source of income. If we do not do this work, we have nothing to eat.”*  *“I used to work as a maid for four families. Since the lockdown declared, I have been told not to come for work. Resultantly, I had no income throughout these periods. My husband is a rickshaw driver, and he started going to work after the two-month lockdown, but he is not getting enough customers and earning.”*  *“My husband is a taxi driver and he used to go to work regularly. Since the lockdown was imposed, he could not find work. During the lockdown, he tried several times but was unable to get enough customers. During that period, he mostly returned home empty handed with zero earnings.”*  *“My husband worked as a daily wage porter in the grain market. He too has not earned anything in the past two months.”*  *“My husband is a rickshaw-puller. We live in a slum with 3 children. Since last year, I have lost 2 jobs, and my husband’s daily income has declined by half. We could not anticipate this situation at all. We had to take loans from others to survive the last few days. All our neighbours are going through the same condition.”*  *“My husband works as a labourer in Dhaka. Due to the nationwide lockdown, his income stopped too. It became burdensome for us to arrange two square meals for our entire family.”* | Aantjes (2022)  AleAhmad (2023)  Azeez E P (2021)  Dogar (2022)  Singh (2022)  Wasima (2022)  Wasima (2022) |
|  |  | 3. Patriarchal society | The pandemic deepened the gendered, de-valued roles that women occupy in society, including through disproportionate burden of increased domestic and unpaid care work as well as diminished economic empowerment. | *“My mother‐in‐law got a fever (implied to Covid‐19 positive)..... Everyone expected me to take care of her and yes, I did it for almost a month….nursed her back to health. It took a toll on me, I also got a fever and felt weak for about 3‐4 days. Nobody pays you for doing this but it is hard work.”*  *“I lost my work and I am seen in the house most of the time. I am called by [in-laws, husband, and child]…asking one or other thing [takes a long pause]…I think my backache started recently, after I lost my rest time.”*  *“I have changed the location of my business, taking care of the child has reduced my ability to work. I don't trade on a regular basis like before.”*  *“He is not violent, but he is very controlling. I sometimes argue with him, but he gets very angry. I try to avoid this, especially at this time. But I feel so claustrophobic (dom bondho lage).”*  *“Women do not always find outside work, but men do. We want to go to work, but there is no work; what to do? There are risks at construction sites; they only allow us to come back after 5 pm. This is very late. If we go there, it will be night when we return.”*  *“We were in lock-down, along with our children. So, my workload increased even further. And financial troubles on top of all. So our routine turned upside down. I got depressed in the early days. I am already a very meticulous person about cleaning, and this doubled or even tripled due to these events. This made me feel mentally distressed. I was crying, and I felt like I was going crazy. I used to have quite a regular life, but now, everything has turned upside down.”*  *Since my husband and I could not go out, there was some stress in the house. As I said, we had a lot of trouble with each other at first. The house being a mess, cooking meals all the time … It’s like you are constantly serving. This makes you nervous in the end. You think they should share the burden. So we were getting a little tense. The whole situation was a powder keg, waiting to blow.”* | Arora (2021)  Bhat (2021)  Boateng-Pobee (2021)  Banerjee (2022)  Barhoi (2024)  Parlak (2023)  Parlak (2023) |
|  |  | 4. Stigma and discrimination | The pandemic amplified existing stigma towards women informal workers by negative framing of them and use of pandemic measures to legitimise discriminatory treatment. | *“In the building where I work, there is a separate lift for maids and drivers. We cannot take the same lift as the residents of the building. This is normal for us but we noticed that people (referring to their employers) thought that poor people like us spread the disease. We maids would have to wear gloves, masks and sometimes a PPE kit given by the employer. It felt so much like untouchability (reference to the discriminating casteist attitude)”*  *“Both my employers were regularly going to office. If you can pick the virus from outside, it would be more likely that I could have picked it from them. But no, they always think that places where poor people live are the ones crammed with insects and diseases.”*  *“They feel that we are carriers of disease…”*  *“I always experienced stigma when I went to shopping places. The perpetrators were both sellers and Page 9/16 buyers. They showed me unwelcoming non-verbal gestures. They made me feel as if I were a coronavirus carrier.”*  *“The police personnel shouted at me, using abusive and dirty words. I was called a pig, carrier of this germ [on the verge of crying, then sobbing, takes a long pause].”*  *“All we want is to have labour rights, to have the right to the emergency aid, not to suffer prejudice from the professionals who serve us. I almost never go to the health unit, a nurse there treated me very bad because she knew how I make my living.”*  *“In June 2020, when the COVID-19 started to spread rapidly in Dhaka city, I used to work at a household in this area. However, when the situation degraded, many residents of that building were tested COVID positive. The employer immediately evicted me since they feared that I might carry the virus from outdoors. All of a sudden, I became jobless without any prior notice.”* | Arora (2021)  Banerjee (2022)  Banerjee (2022)  Belete (2020)  Bhat (2021)  Couto (2022)  Wasima (2022) |
| 2 | Negotiation of risks and resilience | 1. Mixed patterns of belief and protective behaviours | Responses towards COVID-19 reflected a complex mix of beliefs and behaviours ranging from genuine concern for health and fear-driven compliance to scepticism, misinformation and non-compliance. | *“They believed that women who weren’t wearing face masks were not only at high risk but also insensible. So we had to wear face masks not only to protect ourselves but also to attract clients.”*  *“We disinfect our hands. I always take lavender soap and a little bit of bleach with me. I wipe my hands with my towel, and then I eat by myself during the lunch break [...] When I get home, I take a shower and wash the clothes I wore outside.”*  *“I kept on working until the last minute because I don’t have anything to eat (…) I’m not as afraid of the virus as I am of a fine from the police for breaking quarantine.”*  *“I have a bakery business and I along with my employees all work early morning and then during the day for the products to be delivered. When the pandemic hit, I was actually very scared because the sales were going down and I worked extra hard. I wore the mask when working but that was diﬃcult all the time, maybe that is how I got the virus.”*  *“I found out from the news and from people. Through television and news, I was one of the people who did not believe in that, my mouth filled me saying that this was one more mechanism that the government extracted money from corruption. That was a lie.”*  *“There are many people who do not believe, but since I experienced it, I do believe. God left me so that I could tell my life story. God left me so that many people say that Covid does not exist, and I tell them.”*  *“The truth is because I have heard many things about it and every vaccine has the virus. They are dead, but they are there, and I have seen that people have died, and that scares me.”*  *“Doctor, I was afraid of it. Do you remember when files were taken to the radio station, and it was broadcast that 800 people had been given the wrong vaccine? It was broadcast over the radio. Many people had been vaccinated that time.”* | Balampama (2023)  Bossenbroek (2021)  Burgos (2021)  de Silva (2022)  Fabbri (2022)  Fabbri (2022)  Fabbri (2022)  King (2023) |
|  |  | 2. Risk and effect of infection | Occupational and living conditions heightened the exposure of women informal workers to COVID-19 infection. They faced not only physical illness but other consequences including lack of medical attention, job loss and stigma. | *“Even before the lockdown, we had the problem of toilet access. There are toilets, but all are located at the end of the slum. I rarely go there. We often use the deserted space near to the slum. After the lockdown, as all the people stay at home, the toilet becomes very crowded. It is not hygienic and safe, as well. Going to the open space is also difficult now due to the surveillance of many men who stay at home.”*  *“There are some precautions that don’t align with the nature of my work. For instance, wearing face masks, or washing hands regularly during sex is not possible and this is why I feel at risk.”*  *“At the workplace, I am not provided with any protection; travel is also in public transport.”*  *“Farmers do not worry about health measures. They are not interested in us. We come and work the agreed hours and go home. What matters to them is that agricultural work is done well.”*  *“A colleague and I had a little bit of fever and headache, very little cough, for a few days. The manager didn’t let us leave the room on those days so that the ‘clients’ who came wouldn’t see us.”*  *“I stay in a one-bedroom house with two kids, my sister’s two kids and my mother, and other two relatives with limited space to move, as a result, others have to sleep in the lounge and the kitchen. This puts the whole family at risk of contracting the virus as we could not social distance.”*  *“I am forced to reduce my working hours to get home very early. Sometimes I don’t go to work to better supervise the children. Because if you don’t have the necessary means, it’s better to protect yourself than to get sick. Because if you get sick, everyone will be in trouble, especially our children.”*  *“After my husband caught the disease at his workplace and tested positive, I stopped going to work. My test was determined to be positive on nineteenth of April. Both me and my husband were hospitalized.”*  *“I live alone in the city. I did not even tell my parents that I had contracted the disease, even though I was in hospital for three weeks. I am engaged to be married in October. I fear that my engagement would be called off in case anyone gets to know of my illness.”*  *“I could not earn a single penny last month. I was tested positive recently and my employer terminated my employment immediately.”* | Azeez E P (2021)  Balampama (2023)  Bhat (2021)  Bossenbroek (2021)  Burgos (2021)  Mbombo (2022)  Ogando (2022)  Parlak (2022)  Singh (2022)  Wasima (2022) |
|  |  | 3. Exposure to harm | In their struggle to survive during the pandemic, women informal workers took the risk to earn their livelihoods and make ends meet, often under the threat of exploitative conditions and personal danger. | *“For us, who are the street women, a person may come, talks with one of the girls, thinking that he is a client, while he already has a knife and starting to threat to take the phone. You must give it, even with the Mpesa pin [mobile banking system], they will force you to give it then. […] We are really very much at a disadvantage, that’s just it.”*  *“We were nine [women] Kolbars in this village, and now all of us have lost this job. Those eight women, like me, are heads of households and are mothers to several kids. The border closure has left us empty-handed. Three of us decided to go to the border to see if we could cross it but we could not, because there were many guards there. We returned home after a four-hour walk with our loads. To be honest, I was afraid of being killed by the guards.”*  *“I had to change how I charged my clients. This was because most of them complained on being broke because of the pandemic, so I always had to bargain on my charges. I had to agree with them because if I did not, they left for other women and my biggest fear by then was to go back home from work without having earned anything. Before the pandemic, I used to charge them 30,000 TZS but during the pandemic it dropped up to between 10,000 to 5000 TZS.”*  *“They argue that they are doing me a favour by keeping me in employment, as with all the family staying at home, there is no need for a child-minder. My husband has lost his job in the factory, and if my job goes too, how will I feed my two daughters? So I had to agree. They have not even increased the wages for the extra work.”*  *“When the garden reopened, we had to follow new rules. We had to work 1 hour of overtime, and we got Rs. 25 for that hour. They did not give us double wages for overtime work as it is there in the rule.”*  *“Some of my friends had also experienced physical beating by police officers for violating the rule of stay indoors.”*  *“They [the police] screamed that I am increasing their work burden by roaming around…I asked, going to job, is that roaming around?…They were so agitated that my bag was snatched to check, and I was asked to stand there… stood for three hours then they freed me.”*  *“I go from time to time to the moquef...The authorities chase us away. We work secretly so that they don’t see us.”*  *“Some would say you have to sleep with me if you want to work. After sleeping with him, other soldiers would come to chase you away. We were also forced to have sex with law enforcement agents.”*  *“The constant harassment by the police was frustrating. We could not be in the streets at night. The police were using baton sticks to chase us away from the street where we normally found our clients. The situation was challenging, it made it even difficult to access my clients hence no income.”*  *“I had to get money from money landers. Now I just working for them as the interest is 40%. It was a difficult situation for me, since I wasn’t sure when the lockdown would end.”*  *“The harassment was through the beatings with a sjambok (the sjambok or litupa is a heavy leather whip).”*  *“During the first lockdown we were going for fishing, but we were chased away from the lake before we even caught fish. We used to run away from the National Parks’ officials. We used to hide in the bushes with thick vegetation cover and thorns…, we were doing this because we were looking for our families’ survivals. We were just going even if we were being chased away. Running away and hiding became part of our everyday lives.”*  *“One of our peers was called in for a night for 5000 rupees. She was told that there would be only one client. That’s huge money for one client. Usually we don’t go to unknown places like that. But the money was good and she had not got clients the whole week because of lockdown. This money meant that she could get groceries for the next two weeks at least. So she agreed. She was lured into a private farmhouse where seven men raped her till past midnight. She was all battered and shell shocked when she got back.”*  *“I am still in TK10,000 ($120) debt now and have no idea how I will repay it.”*  *‘‘It hasn’t been easy at all for us. We have to smuggle our goods in trucks, pay extra for drivers to transport them unaccompanied or use illegal approved routes. My friend who goes to Nigeria for vegetables introduced me to illegal routes which we use…At one time we went on a boat on the Kano River in Kano State and the waves were very strong. Our boat capsized as the paddler lost control. I was rescued but my friend including some others lost their lives through this.”* | Aantjes (2022)  AleAhmad (2023)  Balampama (2023)  Banerjee (2022)  Barhoi (2024)  Belete (2020)  Bhat (2021)  Bossenbroek (2021)  Crankshaw (2023)  Mavhandu-Mudzusi  (2022)  Mavhandu-Mudzusi  (2022)  Mlambo (2023)  Nhiwatiwa (2023)  Samudyatha (2024)  Wasima (2022)  Wrigley-Asante (2024) |
|  |  | 4. Enabling factors | Factors enabling women informal workers to navigate the pandemic with resilience and resourcefulness. | *“We started giving our number to people and people called us. We survived on it, only phone calls…It made a diﬀerence because people would call us, we would go there and do what we had to do and they would give us our money.”*  *“We felt as if everything had finished, but we soon realized that we needed to pick ourselves up mentally to come out of this trauma. We started motivating each other and with the help of Almighty, family, friends and a few philanthropists, we survived this pandemic.”*  *“After a few weeks of lockdown, we had nothing to keep up with survival, but living with an extended family helped us to survive as we supported each other reciprocally. Secondly, my relatives also demonstrated tremendous generosity in a time of crisis.”*  *“At the outset of COVID, I thought it would end soon, and I could continue my work across borders. But coronavirus is still here and continues to spread. So, I decided to do something else for a living. I knew a woman in a nearby village who is a shoemaker and has been doing this job with her three daughters for seven years. She receives orders from shoe sellers in bigger cities, like Sanandaj, and also from Iraqi Kurdistan.”*  *“Friends are a source of strength and comfort in the bleakness of Covid‐19. When my friend got the infection, we all took care of getting her food and supplies.”*  *“We applied for the ration online with the help of NGO workers. We got it, and it was of great help.”*  *“I am now leading my life by spending a few amounts of money I saved before.”*  *“Everybody (who) have land move from Pattaya to work farm. For me I go home, take care of cow and farm, grow rice, man sampalang (cassava).”*  *“So for me, it was not difficult having to leave them [the children] because I made my own mental shift that the world is not going to end if my son has you know is not paying as much attention to online school at this point in time…so being able to have that mental space where understanding of what I can control and what I can’t control.”*  *“Just being more centred is that I find solace on the farm where I’m working…I think I have grown in my spiritual practice as well, and I have been more consistent working out and I have been able to, I find that massages now actually are therapeutic.”*  *“I have the luck that I put a lot of money also on the side.”*  *“The quarantine was difficult, I found myself desperate, without income. Being with my children, feeling their love helped me to be patient and know that everything passes, we lived one day at a time following the mayor’s orders.”*  *“We managed to do some small piece jobs for those who were going to work to get money and you could manage to get a plate of mealie meal, if you did some laundry, they will give you some bathing and washing soap. It helped a lot.”*  *“I have a grocery store that I run with the support of my family. When the lockdowns and travel restrictions were imposed, I had to ﬁnd a way to sell my goods. That is when I sought my son’s help and opened a WhatsApp account. I would ask my usual clients to send me the grocery list and I would deliver to their house.”*  *“During the pandemic, I faced diﬃculties in purchasing raw materials and conducting my business altogether, and that is when I turned to this women entrepreneur group, one of my fellow businesswomen told me about. I heard about online workshops and tips to survive the pandemic. Overall, it created a very supportive virtual space that wanted to uplift us.”*  *“My friend shared the details of the government direct cash assistance program. I sent my information. After a few hours, I received the message that I was eligible for the program. I thought it might not work but after one and a half week, I received a detailed message on mobile to visit the specific location and collect the money.”*  *“We applied for a loan of 10 thousand liras. They gave us 7 thousand. Also, the state was giving one thousand liras due to the pandemic. We applied for that as well. We were able to breathe again a little bit thanks to these. We are making a living with that money now.”*  *“I stay with my sister who is also a street trader. When we were allowed to return to trading, schools still were not open, so we took turns staying at home with the children. This way we could still do some work.”*  *“The nurse from my treatment centre would send a WhatsApp/SMS message to remind me about the due date for my medication pickup as well as collection of viral load specimen that was due. She would also find out about the safe space where I could meet her for my treatment.”* | Aantjes (2022)  Agha (2022)  Agha (2022)  AleAhmad (2023)  Arora (2021)  Azeez E P (2021)  Belete (2020)  Bishop (2024)  Cadogan-McClean  (2023)  Cadogan-McClean  (2023)  Callander (2022)  Couto (2022)  Crankshaw (2023)  de Silva (2022)  de Silva (2022)  Dogar (2022)  Parlak (2022)  Sithole (2022)  Tshivhase (2023) |
| 3 | Interconnectedness of health and wellbeing stressors | 1. Financial strain | Pandemic undermined financial health by stripping away income sources of women informal workers without adequate provision of aid or relief. This challenge led to compromise in the ability to meet many basic needs. | *“It’s very diﬃcult to see money, it is diﬃcult to aﬀord the home expenses, even the [small] business it’s diﬃcult to make ends meet. I cry every day, until when is this going to end? The President will talk soon, we don’t know if he will give it another thirty days or not.”*  *“In the beginning, the virus looked like a monster who will kill us all. A sense of fear engulfed me. In the beginning, it was too difficult because the sale of my work through the dealer went down. The markets were closed and there was no new order from dealers. In such a situation, we faced big financial challenges, mainly due to the lack of savings.”*  *“We are struggling a lot due to having no income. My mother-in-law sent us some money, and we had to borrow money from a money lender. Everything was going fine, but this situation [referring to COVID-19] put us in debt. We do not know how to return all those money.”*  *“We are in a desperate situation, no food, no medicine, no money. The governments keep announcing schemes but none of these ever reach us.”*  *“…was earning around INR 12 000 per month ($161). This is reduced to now INR 4000 ($53). Now with this reduced amount, I have to feed three stomachs.”*  *“Government give money to people but me no get, my friend no get either. No money for girl work in bar.”*  *““I did not work during the lockdown. [...] I have not received any support because the form was wrongly ﬁlled in. I have suffered psychologically because ﬁnancially well-off people have received help... and I, who needs it, received nothing. I felt injustice, I closed my door, and I did not go out anymore.”* | Aantjes (2022)  Agha (2022)  Azeez E P (2021)  Banerjee (2022)  Bhat (2021)  Bishop (2024)  Bossenbroek (2021) |
|  |  | 2. Physical and mental health | Physical and mental health suffered during the pandemic with reports such as poor hygiene, fatigue, poor sleep, anxiety and depressive symptoms. This experience was connected to other health and wellbeing stressors. | *“The virus and closure of borders have left my brothers-in-law, who were Kolbars, unemployed. They are very angry. They want the home to be quiet when they are home. But we are eight people living in a small house (about 50 m 2). My kids sometimes do local plays at home and make noises or fight. To make them quiet, they lose their temper, shout, hit them or blame me for their improper upbringing. This upsets and distresses me.”*  *“For the last three-four years, I [was]using pads during menstruation. We are not eating enough now. Then how can I think of menstrual pads? As before, I am using cloths now.”*  *“When I encounter all these problems, I feel empty and become hopeless. I am not sure for how long I live like this.”*  *“After lockdown when I restarted my work, I used to have back-to-back sleepless nights…”*  *“It ends up affecting my sleep; I’ve hardly slept since all this began.”*  *“I haven’t slept well, I’m anxious, there were days when I stayed up all night, wondering if I would have money.”*  *“I am an outgoing person. During the lockdown, I had to stay indoors, felt disconnected from my relatives and friends and could not easily have money for WhatsApp bundles. This caused a lot of loneliness and anxiety to me. The whole scenario was frustrating and draining..”*  *“During the lockdown, I felt it was the end of my existence.”*  *“I felt constantly tired. For example, after cleaning a small part of the house or even after dusting a table, I’d have palpitations and would have to sit down and rest. But the fatigue and palpitations would still continue.”* | AleAhmad (2023)  Azeez E P (2021)  Belete (2020)  Bhat (2021)  Burgos (2021)  Couto (2022)  Mavhandu-Mudzusi  (2022)  Oyebamiji (2023)  Parlak (2022) |
|  |  | 3. Shelter and food security | Housing stability and food access were disrupted during the pandemic mainly due to financial hardship. This experience was connected to other health and wellbeing stressors. | *“We started skipping a one-time meal to save our resources and face the crisis.”*  *“I stay silent in front of them because I am afraid of being kicked out since I do not have money to rent a separate house. My family lives in another village. They are very poor, and I cannot ask them for help. My biggest dream is to live with my kids in our own house.”*  *“We may survive the corona virus but we will certainly die of hunger.”*  *“These four months since March was of compromise. We did not have any income. We did not have enough to eat. I compromised with having adequate food too. We cannot eat as before the lockdown. I was able to buy milk for my two children, but now it has become a challenge. They just eat whatever the minimal we prepare for everyone.”*  *“If I do not go back to work within a few days, I will be starved to death.”*  *“I feel so weak and drained out as I eat half of what I used to eat before…already I had the problem of blood [anaemia], now I get severe headaches, backaches, and weakness.”*  *“I’m not afraid of the coronavirus; I’m afraid of not having any money. I don’t know what I’ll do if they throw me out for not paying.”*  *“I have not been able to pay everything. Sometimes, I fall behind in the rent, and food is hard to come by.”*  *“I end up being without any money. I could not even pay the rent, And the landlord have no mercy, she continuously demand rent in foreign currency. She was threatening to evict me.”*  *“In my life, I have seen several difﬁculties but never faced a situation in which I became so hopeless that even I failed to manage the daily food expenses of my family.”*  *“The women fed their children first, and then they themselves would eat if anything was left over, otherwise they would drink water and sleep.”*  *“I was harassed by my house owner for rent. We had lost jobs during lockdown and were running low on our savings. We were not able to pay rent for 4 months.”*  *“I am unable to spend more on food because of reduced earnings, and also it is*  *difficult getting some food items because they are in short supply now. In fact it was horrible during the lockdown but it’s still not very good.”*  *“If we were eating thrice a day before the lockdown, we now eat only once.”* | Agha (2022)  AleAhmad (2023)  Arora (2021)  Azeez E P (2021)  Belete (2020)  Bhat (2021)  Burgos (2021)  Fabbri (2022)  Mavhandu-Mudzusi  (2022)  Mustafa (2021)  Ogando (2022)  Samudyatha (2024)  Wrigley-Asante (2024)  Zulfiqar (2022) |
|  |  | 4. Interpersonal tension | Pandemic stress negatively affected familial and workplace relationships. This experience was connected to other health and wellbeing stressors. | *“There’s an example like this, someone may come to hit you here, nobody helps you, here nobody helps you [in] this time of corona but before they didn’t accept it but now they let you hit you well until you leave, yes because of all that money.”*  *“For people like us there are no laws, no benefits to protect us and before any virus affects us, either hunger or our husbands will kill us.”*  *“There was no money and no ration at home. I could not go to work, and my husband used to beat me every day at that time. The lack of work at that time impacted our domestic affairs.”*  *“I was already facing verbal abuse and physical hitting for the last 12 years… now as daru (alcohol) is not available and he is not having regular work [as a painter], he feels frustrated.”*  *“At the market I always fight with the women over their careless attitude towards the protective measures so I have even warned them not to handle my child or even give him some of their food when eating. When I tell them that they get angry, but I don’t care, I have to protect my child.”*  *“I’m very irritated, impatient with my children complaining about coming home without money, without food. Having to ask my mother and her throwing it in my face.”*  *“My husband is a chain smoker, he needs two packets of cigarettes daily. I managed it for one and a half week but then it was difficult for me to manage such amount of money every day. My husband asked for money, but I refused many times, and told him that we are going short of money, and it was difficult to manage the household expenditures. Gradually, he became annoyed and aggressive. He attempted three times to beat me, but I luckily managed to escape.”*  *“Yes, you know I got a new boyfriend and I have been staying at his home but the biggest challenge with him is that when he doesn’t get money, he transfers the anger to me and beats me up.”*  *“People cannot tolerate anything. There are times we shout and get angry at even to our own children. That’s pretty bad. I am nervous and angry. Because it’s always the same things, routines. There is nothing extra to do. People get tired after a while. You don’t feel like doing anything. We can’t go anywhere. We can’t even accept guests at home.”*  *“My husband drinks and creates frequent problems in the house. This increased during the lockdown. It was difficult for me to go out for work during lockdown, because he was staying at home all the time. He would grow suspicious, wondering where I was going when everybody was staying home. It was very frustrating. Neither was he getting anything to feed the children, nor was he allowing me to do something.”* | Aantjes (2022)  Banerjee (2022)  Barhoi (2024)  Bhat (2021)  Boateng-Pobee (2021)  Couto (2022)  Dogar (2022)  King (2023)  Parlak (2022)  Samudyatha (2024) |
|  |  | 5. Barriers to healthcare | The strain on healthcare system combined with other factors such as financial hardship and fear of contracting COVID-19 infection left women informal workers unable or unwilling to seek healthcare. This experience was connected to other health and wellbeing stressors. | *“Since this disease started spreading, I am really worried about my family. If anyone from the slum gets infected with this virus, it will spread everywhere in the slum because maintaining distance is impossible. Houses are very close. I am worried because we do not have money for the treatment if anybody gets the disease.”*  *“If I go to a government centre for test without any sign of fever or doctor's prescription, they will drive me away. People like me cannot afford to go to private facilities. If they are so concerned, they should pay for it. I don't know what to do.”*  *“I had my hysterectomy six weeks before lockdown. However, I was unable to go for any follow-up check-up. There was no way to reach out for further article consultations. I feel tired and backache is severe…but nothing can be done without money.”*  *“I no longer take medication for my heart disease ... I ﬁnished the last box before Ramadan, and I no longer bought it. [...] I stopped it for two reasons: I am afraid to go to the doctor, and I do not have the money to buy the medicines.”*  *“Sex workers, we don't get provided health insurance, what will we do if we get infected? That's a big ﬁnancial risk and stress for us.”*  *“Because I could not afford the private clinic expenses, we mostly relied on old medication, but for me it was very hard to purchase medicines.”*  *“And with health services, I try to avoid it because making an appointment is worth money and they do not examine anything. If it is an emergency, I go to an emergency center, but I do not go all the time because it also costs.”*  *“I have missed my appointments to the clinic at BHESP. I was supposed to go collect my ARVs but now with the lockdown, how will I go to collect them? I cannot visit the public health facility because of stigma and discrimination.”*  *“In this current situation we no longer have anywhere to get condoms.”*  *“Right now, we fear going to health facilities because there is COVID, and we might get it from there; besides most people are now looking at COVID-19 as a priority and not these other things like family planning.”*  *“I am three months pregnant but I could not afford my checkup expenses due to my business failure. I am extremely depressed.”*  *“I was in the sixth month of pregnancy when the lockdown was announced. If I had some money, I would go for check-ups, otherwise I would stay at home. Only when I was unable to bear the pain, I would see the doctor. I was experiencing shortage of blood, for which I had been advised to take an injection. But we could not afford that. I developed complications because of that and was operated upon in the eighth month only. I try to not remember that time.”* | Azeez E P (2021)  Banerjee (2022)  Bhat (2021)  Bossenbroek (2021)  Callander (2022)  Dogar (2022)  Fabbri (2022)  Gichuna (2020)  King (2023)  King (2023)  Mustafa (2021)  Vyas (2023) |
|  |  | 6. Health behaviour | Pandemic influenced behaviour towards substance use, sexual behaviour and other health behaviours such as adherence to medicine. This experience was connected to other health and wellbeing stressors. | *“Bhang would cost me 50ksh (0.50 USD), which right now goes to food. I also do not drink [alcohol] unless I ﬁnd a friend drinking and I join in. I would like to think that I am reforming and I have become wiser. Even when things come back to normal, I do not think I will be spending as much money on Bhang as I did before. I was misusing money. I used to spend 300ksh (3 USD) on alcohol and bhang in a day and nowadays I go a week without getting as much. It has been truly eye-opening.”*  *“I cannot use substance daily because I don’t have money to buy it. I feel dizzy when I do not chew khat. I have now started to hate myself and sometimes I wish to die.”*  *“I tried to stop consuming these substances, but it is not as such simple and I could not cope with health symptoms such as moodiness, irritability and severe headache. I get worried when I do not have money to buy substances. I prefer to use substance instead of experiencing dizziness. I am now thinking that I can be only cured from addiction with holy water if it is God’s will.”*  *“I am living with HIV. When some clients demand unprotected sex, I advise them not to do it. But, if they do not accept my advice, I offer them unsafe sexual service because I cannot survive in this pandemic period unless I make some money. Previously, I did not practice unprotected sex in order to protect my clients from HIV virus but after the coronavirus outbreak, I could not protect some of my clients from HIV infection as I do not have any other option for survival.”*  *“I think I've been using more because of isolation, and there's not as much support. Because normally there's [Alcoholics Anonymous] groups, [Narcotics Anonymous] groups, things like that, and now there's like nothing.”*  *“I drink a lot to face life and clients, but during the quarantine, without a client I drank more, I stopped eating to drink. Also because I had little food and left it for my children.”*  *“Normally I could not have sex without a condom, but the prevailing situation forced me to. I needed the money and some clients I met were insisting on having unprotected sex. Also, having unprotected sex risky as it were, I was able to get more money from clients.”*  *“What else can l do during this pandemic? If I doesn’t drink I will die of stress. Who knows when the pandemic will end.”*  *“During lockdown, I did not have any means of earning. The number of customers was low. If you don’t have money and if the customer is offering you more, then you have no choice but to agree for condom-less sex.”* | Babu (2024)  Belete (2020)  Belete (2020)  Belete (2020)  Callander (2022)  Couto (2022)  Mavhandu-Mudzusi  (2022)  Nyabeze (2022)  Samudyatha (2024) |
| 4 | Variable experiences across social locations | 1. Sex workers | Sex workers experienced compounded vulnerability during the pandemic due to stigma attached to their work and risk of sexually transmitted diseases. Experiences of sex workers varied based on characteristics such as their race and work venue. | *“The government considers sex workers as a social disease, and because of this stereotype the government does not show any interest in providing economic support for us during this pandemic period.”*  *“The government has announced a 50% reduction of housing rent due to the coronavirus pandemic. Many people have benefitted from this. As opposed to this announcement, many house owners have doubled the monthly rental price on commercial sex workers in order to push us to leave their houses. We have reported this crime at the police station and local authority once. None of these institutions have shown interest to investigate our complains instead they consider us as the enemies of social order.”*  *“So sex workers of colour can and have always had to, they're at the margins, it's hard to break in more because the niche is more for more white women, white males.”*  *“Our profession has never been recognized, even politicians come to us, they want to pay anything and they don’t organize to have our rights. If we didn’t have it before,*  *imagine now in the pandemic.”*  *“It was hard; my neighbours helped me. They sometimes gave me food to eat for almost three days, I did not tell them what I do for a living to avoid being judged and shunned by society, I had to lie and say I was laid off from work owing to COVID-19.”*  *“Let me tell you one thing why many of us don’t use condoms, we don’t have money, and when you meet a client who offers to give you more money than you usually get, you have sex without protection even when you don’t know his HIV status.”*  *“I got grocery kits from here during the lockdown. My neighbours were not particularly happy with it. They asked me repeatedly–how is that you are getting grocery kits while we are not? I told them that I got it from an NGO where I was working. As soon as they heard it, they also wanted to join the NGO! How can I tell them that I was a sex worker and that is why I am a member of this NGO? Finally I ended up sharing some of my grocery with them so that they would stop asking questions.”* | Belete (2020)  Belete (2020)  Callander (2022)  Couto (2022)  Mlambo (2023)  Nyabeze (2022)  Samudyatha (2024) |
|  |  | 2. Migrant women | Migrant women informal workers faced compounded vulnerability during the pandemic from factors such as xenophobia and legal precarity. Being away from their initial homes brought different challenges and coping strategies. | *“Suppose you migrate to a foreign country in search of a better life and job, get married and settled there and have children. How would you feel if you had to suddenly leave everything there and come back because you lost your job and don’t have any money? It is the same for us.”*  *“City life is tough, it is full of struggle but going back means that all that struggle was for nothing. Our years here and all the hard work meant nothing…..My gaon (village) is not my home…back in the village everyone knows me and I will have to stay with my in‐laws, follow all the traditions and will have no scope for employment.”*  *“But there were no trains. The government should have supported us going back home. Later, when there were trains, we heard the charge is high.”*  *“Ummm! [sic], Since I am a foreigner, we are always removed from our selling point by local women. It is hard, we just move and look for another selling point.”*  *“Locals [were] mobilising to chase [evict] away foreigners [migrants] out of their rental homes…after the lockdown.”*  *“They (xenophobes) have always threatened us by making false accusations about our presence here,…we fear for our lives including being raped with no case [recourse].”* | Arora (2021)  Arora (2021)  Azeez E P (2021)  Mapuranga (2021)  Muswede (2022)  Muswede (2022) |
|  |  | 3. Identity categories | Pandemic affected women informal workers unequally, with overlapping social identities such as religion, race, ethnicity, class, caste and age shaping their experiences. | *“I have accumulated debts because I’m hardly earning any money yet I have rent to pay. I have children to feed and I am a single parent. I think I might have been depressed because of these.”*  *“I go to work outside at an Assamese people’s house. They give us more wages than the tea garden. They give lunch, tea, and biscuits too, plus Rs. 200,which is enough for me. This is more profitable for me. But they give me to eat separately with separate utensils. They do not consider me Assamese, and one of them; they call me Adivasi.”*  *“Permanent workers received Rs. 500 as help during that period. But, faltu received nothing. Don’t we eat food? Faltu did not receive ration during the lockdown as we were not working at that time.”*  *“You know just thinking about it is heart-breaking, I am a new mom that has to breastfeed. There were times when my breasts wouldn’t have any milk because I did not eat too. What really helped me survive lockdown is cooking imbila (maize porridge) and I would drink it every time I would get hungry, level 5 felt like a never-ending nightmare, I don’t want to go back there.”*  *“I am in my 50’s. I got infected with COVID19. I had to stop coming to work because I was scared to expose myself. I do not have a shelter, so I am in the open; I did not want to get sick.”* | Babu (2024)  Barhoi (2024)  Barhoi (2024)  Mbombo (2022)  Sithole (2022) |
|  |  | 4. Positive experiences | Despite challenges, some women informal workers adapted positively during the pandemic, particularly those with access to skills and resources such as family support and healthcare provision. | *“Thanks to the lockdown, I got to be with both of them and my husband, who was also a government teacher and had to be home since schools were closed.”*  *“When the lockdown was enforced, most people could not come outside their homes. Because of this situation, some customers who had my contact called me on phone to deliver foodstuﬀs such as cassava, plantain, vegetables and some provisions [groceries, emphasis added] to them in their homes. Also, sobolo leaves (hibiscus leaves, also called bissap) were in great demand because it was popular for treating the COVID-19 virus. Because we [petty market traders] were allowed to work at the market during the lockdown, I also served customers in their homes as they could not come in-person to the market to buy some of these food items. My work activities, therefore, increased in terms of the customers I had to attend to as well as my overall work output.”*  *“I started making masks because everyone was making masks. I had a sewing machine. I literally did not know how to sew but I quickly figured it out, so I started selling masks so that was a good thing.”*  *“Service providers came to the community to provide the services we required since it was difficult for us to visit the healthcare facilities due to the COVID-19 restrictions. An arrangement was made for us to meet the healthcare workers to access our ARV medicines, get condoms. The clinic would call to find out the preferred/convenient place to deliver the medicines. This arrangement ensured privacy and was very convenient since movement was a challenge.”*  *“The nurses and other community workers were supportive and brought services closer to our homes. This made the situation better and easier for me, in that I did not need to look for transport to go to the health facilities.”* | Abrefa Busia (2023)  Abrefa Busia (2023)  Cadogan-McClean  (2023)  Tshivhase (2023)  Tshivhase (2023) |
